# Sensor Selection for Decentralized Large-Scale Multi-Target Tracking Network

**DOI:** 10.3390/s18124115

**Published:** 2018-11-23

**Authors:** Feng Lian, Liming Hou, Bo Wei, Chongzhao Han

**Affiliations:** Ministry of Education Key Laboratory for Intelligent Networks and Network Security (MOE KLINNS), School of Electronics and Information Engineering, Xi’an Jiaotong University, Xi’an 710049, China; hliming2017@stu.xjtu.edu.cn (L.H.); weibo18291927630@163.com (B.W.); czhan@xjtu.edu.cn (C.H.);

**Keywords:** sensor selection, multi-target tracking, labeled random finite set, decentralized sensor network, error bound

## Abstract

A new optimization algorithm of sensor selection is proposed in this paper for decentralized large-scale multi-target tracking (MTT) network within a labeled random finite set (RFS) framework. The method is performed based on a marginalized δ-generalized labeled multi-Bernoulli RFS. The rule of weighted Kullback-Leibler average (KLA) is used to fuse local multi-target densities. A new metric, named as the label assignment (LA) metric, is proposed to measure the distance for two labeled sets. The lower bound of LA metric based mean square error between the labeled multi-target state set and its estimate is taken as the optimized objective function of sensor selection. The proposed bound is obtained by the information inequality to RFS measurement. Then, we present the sequential Monte Carlo and Gaussian mixture implementations for the bound. Another advantage of the bound is that it provides a basis for setting the weights of KLA. The coordinate descent method is proposed to compromise the computational cost of sensor selection and the accuracy of MTT. Simulations verify the effectiveness of our method under different signal-to- noise ratio scenarios.

## 1. Introduction

With the development of communication and information fusion technologies, multi-target tracking (MTT) [1] based on sensor network becomes a new research hotspot. In general, the sensor networks are divided into two main categories according to their structure: one is centralized network and the other is decentralized network. Compared with the centralized network, the decentralized network is more widely concerned because of its parallelism, flexibility, robustness, scalability, anti-interference and fault tolerance, et al. In most practical applications, due to the limitations of communication bandwidth, energy consumption, computational cost, storage space et al, not all sensors in a network can be activated to observe targets at the same instant. As a result, the problem of sensor selection arises from this, which actually belongs to a branch of sensor management [2]. Although some research results [3,4,5,6,7,8] have been proposed for it, none of them jointly consider the uncertainty of target number and data association.

In the past two decades, random finite set (RFS) [9] based MTT has attracted extensive attention. By the use of RFS, MTT is described as a Bayesian estimation of state and observation sets. RFS filtering has been developed from the primal probability hypothesis density (PHD) [10,11,12], cardinalized PHD [13,14] and multi-Bernoulli [15,16] filters to the latest δ-generalized labeled multi-Bernoulli (δ-GLMB) filter [17,18,19]. The advantage of the latter is its conjugacy and track formation. Nevertheless, the number of components involved in the GLMB density increases exponentially with the recursion. Therefore, two approximation methods, the LMB and Marginalized δ-GLMB (Mδ-GLMB) [20,21,22], are subsequently proposed to reduce the computational cost of GLMB. They are also more suitable for multi-sensor scenarios. [23] has shown that the filtering accuracy of Mδ-GLMB is close to δ-GLMB and the two are significantly superior to LMB in the scenarios of low signal-to-noise ratio (SNR).

Besides the δ-GLMB conjugate prior, the Poisson multi-Bernoulli mixture (PMBM) filter [24,25] and multi-Bernoulli mixture (MBM) filter [25] are also conjugate priors. Track formation in the (P)MBM formulation can also be attained using RFS of trajectories [26].

In recent years, although the RFS-based methods have been used to control the position of one or several mobile sensors for MTT [27,28,29,30,31,32,33,34], none of them refer to the sensor network. Actually, in many cases, the sensors in a network are immobile. Instead, the problems of structure, constraints, node selection and so forth become rather important especially for a large-scale sensor network.

As a result, this paper focuses on the emerging problem of sensor selection for decentralized large-scale MTT networks. A new optimization algorithm for sensor selection is proposed based on the Mδ-GLMB filter. In the proposed method, the fusion of local multi-target posterior densities is carried out by using the rule of weighted Kullback-Leibler average (KLA) [23].

The main contributions of our method includes four aspects. First, the sensor selection is described as a constrained optimization problem with a Bayesian recursion of labeled multi-target RFS. A new metric, named as the label assignment (LA) metric, is proposed to measure the distance for two labeled sets. The lower bound of LA metric based mean square error (MSE) between the labeled multi-target state set and its estimate is treated as the optimized objective function of sensor selection. The bound is derived by the information inequality to RFS measurement [35]. The detailed proofs for the LA metric and its lower bound are presented in the appendices. Second, the normalized weights of the KLA rule are set according to the proposed bound. Third, both the sequential Monte Carlo (SMC) [10,36] and Gaussian mixture (GM) [11,12,13,14,15,16] implementations for the bound are presented. Fourth, because the computational cost of selection optimization increases with sensor number in the form of combination explosion, a sub-optimization method called coordinate descent [37] is proposed to compromise the computational cost and tracking accuracy.

The simulation results show that when the sensors in a decentralized large-scale network have different observation performance, 1) the MTT accuracy of our method is much better than that of the Cauchy-Schwarz (CS) divergence based methods [31,32]; 2) compared with the genetic algorithm [38], the coordinate descent method significantly shortens the calculation time of sensor selection; 3) the GM implementation of the bound is obviously faster than its SMC implementation.

## 2. Mathematical Background

### 2.1. Labeled RFS and Mδ-GLMB

In this paper, the unlabeled and labeled variables are, respectively, represented by the italics and bold. For example, the unlabeled state, measurement and their sets are noted as x, z, X and Z; the labeled state and its set are noted as x=(x,ℓ) and X, where ℓ is the discrete label of x. Let ℒ(X), |X| and X×L denote the label set, cardinality and space of X, where X and L are the spaces of the unlabeled state and label.

The state estimates of single-target and multi-targets derived from a measurement set Z are both the functions of Z. To make this clearer, they are, respectively, noted as $\hat{x}(Z)$ and $\hat{X}(Z)$. $\hat{x}(Z)$ and $\hat{X}(Z)$ are their labeled versions.

Let $\delta_{Y}(X)$, $1_{Y}(X)$ and $p^{X}$ denote the functions of generalized Kronecker, inclusion indicator and multi-object exponential,

(1) $$\delta_{Y}(X)=\left\{ \begin{array}{l} \begin{array}{cc} 1, & \mathrm{if}\;X=Y \end{array}\\ \begin{array}{cc} 0, & \mathrm{otherwise} \end{array} \end{array}\right.$$

(2) $$1_{Y}(X)=\left\{ \begin{array}{l} \begin{array}{cc} 1, & \mathrm{if}\;X\subseteq Y \end{array}\\ \begin{array}{cc} 0, & \mathrm{otherwise} \end{array} \end{array}\right.$$

(3) $$p^{X}=\left\{ \begin{array}{l} \begin{array}{cc} \prod_{x\in X}p(x), & X\neq \emptyset \end{array}\\ \begin{array}{cc} 1, & X=\emptyset \end{array} \end{array}\right.\;$$

where $1_{Y}\left(\left\{x\right\}\right)$ is abbreviated as $1_{Y}(x)$. Furthermore, if $1_{Y}(X)=1$, then let Y−X denote the complementary set of X in Y. Y−{x} is abbreviated as Y−x.

For any real-valued function b(X) of X, its set integral ∫b(X)δX is defined as

(4) $$\int b(X)\delta X=\sum_{n=0}^{\infty }\frac{1}{n!}\sum_{\ell_{1:n}\in L_{n}}\int_{X_{n}}b\left(X_{n}\right)dx_{1:n}\;$$

where $x_{1:n}=x_{1},\ldots ,x_{n}$ and $\ell_{1:n}=\ell_{1},\ldots ,\ell_{n}$, $X_{n}=\left\{x_{1:n}\right\}$ is a n-element labeled set, $X_{n}$ and $L_{n}$ are the spaces of $X_{n}$ and $\ell_{1:n}$.

If X is a Mδ-GLMB RFS, then its density is described as [22]

(5) $$\pi (X)=\Delta (X)\sum_{I\in ℱ(L)}\delta_{I}\left(ℒ(X)\right)\omega_{I}p_{I}^{X}\;$$

where $\Delta (X)=\delta_{\left|X\right|}\left(\left|ℒ(X)\right|\right)$ is a distinct indicator for the labels of X, I∈ℱ(L) is a label set in the collection ℱ(L) of finite subsets of L, the weight $\omega_{I}$ is the existing probability of the label set I, $p_{I}(x)$ is the density of x involved in I. The Mδ-GLMB density is abbreviated as $\pi ={\left\{\left(\omega_{I},p_{I}\right)\right\}}_{I\in ℱ(L)}$ and its cardinality distribution is

(6) $$P\left(|X|=n\right)=\sum_{I\in {ℱ}_{n}(L)}\omega_{I}\;$$

where ${ℱ}_{n}(L)$ is the collection of n-element subsets of L.

### 2.2. Information Inequality to RFS Measurement

Let $\hat{x}\left(Z_{m}\right)$ be an unbiased estimate of x derived from an m-element measurement set $Z_{m}$ and $f\left(x,Z_{m}\right)$ be a joint density over the space $X_{1}\times {\mathbb{Z}}_{m}$. Assuming that regularity conditions hold and $\partial^{2}\log f\left(x,Z_{m}\right)/\partial x^{i}\partial x^{j}$ exists, the information inequality to RFS measurement is [35]

(7) $$\begin{array}{cc} \int_{{\mathbb{Z}}_{m}}\int_{X_{1}}f\left(x,Z_{m}\right){\left(x^{l}-{\hat{x}}^{l}\left(Z_{m}\right)\right)}^{2}dxdz_{1:m}\geq {\left[J_{m}^{-1}\right]}^{l,l}, & l=1,\ldots ,L \end{array}\;$$

where $z_{1:m}=z_{1},\ldots ,z_{m}$, L is the dimension of x, $x^{l}$ and ${\hat{x}}^{l}\left(Z_{m}\right)$ are the lth components of the vectors x and $\hat{x}\left(Z_{m}\right)$, $J_{m}$ is the L×L Fisher information matrix (FIM) given |Z|=m,

(8) $$\begin{array}{cc} {\left[J_{m}\right]}^{i,j}=-E_{f}\left[\frac{\partial^{2}\log f\left(x,Z_{m}\right)}{\partial x^{i}\partial x^{j}}\right]=-\int_{{\mathbb{Z}}_{m}}\int_{X_{1}}f\left(x,Z_{m}\right)\frac{\partial^{2}\log f\left(x,Z_{m}\right)}{\partial x^{i}\partial x^{j}}dxdz_{1:m}, & i,j=1,\ldots ,L \end{array}\;$$

(7) holds with equality if and only if $f\left(x,Z_{m}\right)$ satisfies the distribution of exponential family.

### 2.3. A New Metric for Labeled RFS

It is well known that the optimal sub-pattern assignment (OSPA) metric [39,40,41] has been extensively used to measure the distance for two unlabeled sets. Although the OSPA metric could also measure differences in the set labels, it may not be very appropriate for the labeled RFS in some application scenarios. As a result, a new metric between two labeled sets X and Y of order 1≤p≤∞ with cut-off c>0 is proposed as follows.

(9) $${\bar{d}}_{p}^{\left(c\right)}\left(X,Y\right)=\left\{ \begin{array}{l} \begin{array}{cc} 0 & |X|=|Y|=0 \end{array}\\ \begin{array}{cc} {\left(\frac{\sum_{\ell \in ℒ(X)\cap ℒ(Y)}d^{\left(c\right)}{\left(x_{\ell },y_{\ell }\right)}^{p}+c^{p}\left(\left|ℒ(X)\cup ℒ(Y)\right|-\left|ℒ(X)\cap ℒ(Y)\right|\right)}{\left|ℒ(X)\cup ℒ(Y)\right|}\right)}^{\frac{1}{p}} & |X|+|Y|>0 \end{array} \end{array}\right.\;$$

where ℒ(X) and ℒ(Y) are the label sets of X and Y, $x_{\ell }\in X$ and $y_{\ell }\in Y$ are the unlabeled elements corresponding to the label ℓ, |ℒ(X)∩ℒ(Y)| and |ℒ(X)∪ℒ(Y)|−|ℒ(X)∩ℒ(Y)| respectively indicates the number of elements in X and Y which have the same and different labels,

(10) $$d^{\left(c\right)}\left(x_{\ell },y_{\ell }\right)=\min\left(c,\left\|x_{\ell }-y_{\ell }\right\|\right)\;$$

denotes the 2-norm of $x_{\ell }$ and $y_{\ell }$ cut off at c>0.

See Appendix A for the proof that the ${\bar{d}}_{p}^{\left(c\right)}\left(X,Y\right)$ is a metric.

The proposed metric, which is named as the LA metric by us, is a different metric than the OSPA. A physical meaning about the LA metric between two labeled sets X and Y is explained as follows. For all x∈X and y∈Y, if x has the same label as y, then x is paired with y and a ‘location’ error $d^{\left(c\right)}\left(x,y\right)$ between them is involved in the metric; otherwise, x is unpaired with y and a ‘penalty’ error c for them is involved in the metric.

The most significant difference between the OSPA metric and the LA metric is: In the OSPA metric, the elements of X are paired with the elements of Y depending on the optimal assignment distance of their unlabeled versions. In contrast, in the LA metric, the elements of X are paired with the elements of Y completely depending on their labels.

Take the MTT with the labeled RFS state for example. The calculation of the OSPA error may pair an estimate with a state due to the rule of optimal assignment even if they have different labels. Instead, the calculation of LA error prohibits this kind of pairing even if their unlabeled states are sufficiently close to each other. As a result, the LA metric involves not only the estimation error arising from the target number and individual states as the OSPA metric but also the additional estimation error arising from the labels. In this sense, the LA metric is more demanding than the OSPA metric for measuring the error between the labeled state set and its estimate.

## 3. Problem Formulation

To simplify the formulas, the time index is omitted and the subscript ‘+’ is used to indicate the predicted density.

Multiple targets independently move in region A with random birth and death. The multi-target states are modeled as a labeled RFS X. The dynamic of a single state x=(x,ℓ)∈X is described by the survival probability $p_{s}(x)$ and transition density $f\left(x|x^{\prime }\right)\delta_{\ell^{\prime }}(\ell )$. The dynamic of multi-target states is described by the transition density $f\left(X|X^{\prime }\right)$. Here $x^{\prime }=\left(x^{\prime },\ell^{\prime }\right)$ and $X^{\prime }$ are, respectively, the state and state set at the last time.

**Figure 1:**
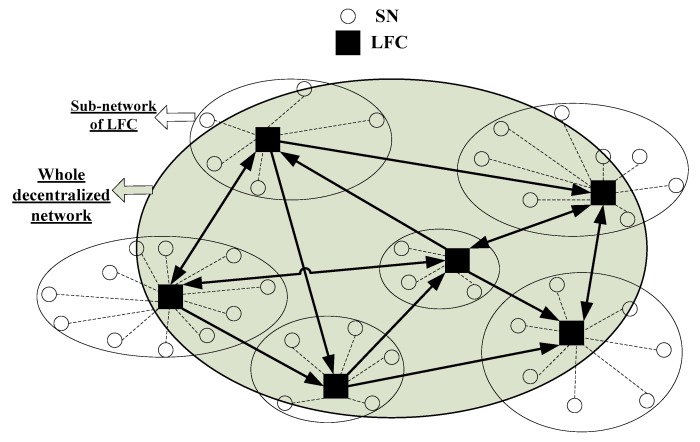
Diagram for decentralized sensor network. ○ and ■ denote SN and LFC, solid lines with arrows denote directed connections between LFCs, the dotted lines denote connections between LFC and its subordinate SNs.

Targets are observed by the decentralized sensor network shown in Figure 1. The network is composed of sensor nodes (SNs) and local fusion centers (LFCs). Each SN receives measurements and communicates with its superior LFC. Each LFC receives the measurements, conducts data processing and storage, communicates with the other LFCs connected to it and manages its subordinate SNs.

The network structure is completely described by a topological graph with parameter {N,C,A}, where N and C are the label sets of SN and LFC, A⊆C×C is the set of directed connections between LFCs. If the LFC j can receive data of the LFC i, then (i,j)∈A. Let $C^{j}=\left\{i\in C:\left(i,j\right)\in A\right\}$ be the label set of the LFCs connected to the LFC j (including itself) and $N^{j}$ be the label set of the SNs belonging to the LFC j. Each SN only belongs to one LFC, which indicates $\cap_{j\in C}N^{j}=\emptyset$ and $\cup_{j\in C}N^{j}=N$.

The most significant difference between the decentralized network and the centralized or hierarchical network is that the former has no global fusion center connected to all SNs or all LFCs. The network structure remains unchanged and all measurements are synchronized during the monitoring period.

For the SN s∈N, it may receive clutter and target measurement or miss the detection. The measurements are modeled as a RFS $Z^{s}$ over the space ${\mathbb{Z}}^{s}$. $z^{s}\in Z^{s}$ is a single measurement. Clutter is modeled as a Poisson RFS with intensity $\kappa^{s}\left(z^{s}\right)$, and

(11) $$\lambda^{s}=\int_{{\mathbb{Z}}_{1}^{s}}\kappa^{s}\left(z^{s}\right)dz^{s}\;$$

is the clutter rate.

The multi-target likelihood of the SN s is obtained from [9] as

(12) $$g^{s}\left(Z^{s}|X\right)=e^{-\lambda^{s}}{\left[\kappa^{s}\right]}^{Z^{s}}\sum_{\theta^{s}\in \Theta^{s}}{\left[\psi_{Z^{s}}^{s}\left(\cdot ;\theta^{s}\right)\right]}^{X}\;$$

where

(13) $$\psi_{Z^{s}}^{s}\left(x;\theta^{s}\right)=\delta_{0}\left(\theta^{s}(\ell )\right)\left(1-p_{d}^{s}(x)\right)+\left(1-\delta_{0}\left(\theta^{s}(\ell )\right)\right)\frac{p_{d}^{s}(x)g^{s}\left(z_{\theta^{s}(\ell )}^{s}|x\right)}{\kappa^{s}\left(z_{\theta^{s}(\ell )}^{s}\right)}\;$$

where $p_{d}^{s}(x)$ and $g^{s}\left(z^{s}|x\right)$ are the single-target detection probability and likelihood, $\Theta^{s}$ is a collection of association mapping $\theta^{s}:ℒ(X)\to \left\{0,1,\ldots ,\left|Z^{s}\right|\right\}$. $\theta^{s}(\ell )>0$ or $\theta^{s}(\ell )=0$ indicates that the track ℓ∈ℒ(X) generates a measurement $z_{\theta^{s}(\ell )}^{s}\in Z^{s}$ or be missed. Each track at most generates one measurement and each measurement is at most generated by one track, which indicates that $\ell =\ell^{\prime }$ if $\theta^{s}(\ell )=\theta^{s}(\ell^{\prime })>0$. The number of association hypotheses is [42]

(14) $$\chi_{\left|ℒ(X)|,|Z^{s}\right|}=\sum_{i=0}^{\min(|ℒ(X)|,|Z^{s}|)}\frac{\left|ℒ(X)\right|!\cdot \left|Z^{s}\right|!}{\left(\left|ℒ(X)\right|-i\right)!\cdot \left(\left|Z^{s}\right|-i\right)!\cdot i!}\;$$

Due to some restrictions, only part of SNs involved in the sub-network of each LFC can be activated to observe targets at each scan. Assume that the multi-target likelihoods of all the SNs in the network are independent of each other given the labeled state set X. Algorithm 1 presents the steps of sensor selection and MTT for the LFC j∈C under a Bayesian framework. Note that the sequence of measurement sets up to the last time is omitted here for simplifying the formulas of Bayesian recursion.

**Algorithm 1.** Sensor selection and MTT for the LFC j.**1. Prediction:** Calculate the current predicted density by $\pi_{+}^{j}(X)=\int f\left(X|X^{\prime }\right){\bar{\pi }}^{j}(X^{\prime })\delta X^{\prime }$, where ${\bar{\pi }}^{j}(X^{\prime })$ is the fused density at the last time;**2. SN selection:** Select the SN subset $S^{j}\subseteq N^{j}$ and receive a collection of their measurement sets $Z^{S^{j}}$;**3. Update:** Calculate the current posterior density by $\pi^{j}\left(X|Z^{S^{j}}\right)=\frac{\prod_{s\in S^{j}}g^{s}\left(Z^{s}|X\right)\pi_{+}^{j}(X)}{\int \prod_{s^{\prime }\in S^{j}}g^{s^{\prime }}\left(Z^{s^{\prime }}|X\right)\pi_{+}^{j}(X)\delta X}$ and then transmit the density to the LFCs connected to the LFC j;**4. Fusion:** Receive the posterior densities from the LFC set $C^{j}$ and then calculate the fused density by the weighted KLA rule ${\bar{\pi }}^{j}(X)=\frac{\prod_{i\in C^{j}}{\left[\pi^{i}\left(X|Z^{S^{i}}\right)\right]}^{\alpha^{j,i}}}{\int \prod_{i^{\prime }\in C^{j}}{\left[\pi^{i^{\prime }}\left(X|Z^{S^{i^{\prime }}}\right)\right]}^{\alpha^{j,i^{\prime }}}\delta X}$, where $\alpha^{j,i}$ ($i\in C^{j}$) is the preset normalized weight;**5. State extraction:** Extract the current state estimate from ${\bar{\pi }}^{j}(X)$ as the output. Go to Step 1.

**Remark** **1.** *It can be seen from Steps 3 and 4 of Algorithm 1 that in this network, each LFC only communicates once with the LFCs connected to it per recursion. Because of this, the consensus iteration [43,44] of each LFC cannot be carried out since each LFC has to communicate with other LFCs more than once in the iterative process. Finally, the consensus fusion [23] over the entire decentralized sensor network cannot be achieved. As a result, the multi-target estimates outputted by different LFCs may be different. In fact, this character is consistent with most practical application systems.*

The SN selection in Step 2 of Algorithm 1 is described as the following constrained optimization problems:

(15) $$\begin{array}{l} {\left[S^{j}\right]}^{*}=\underset{S^{j}\subseteq N^{j}}{\arg\min/\max}\;\vartheta^{j}\left(S^{j};\pi_{+}^{j}\right)\\ s.t.\left\{ \begin{array}{l} \begin{array}{cc} \gamma_{i}^{j}\left(S^{j};\pi_{+}^{j}\right)\geq 0 & i=1,\ldots ,l \end{array}\\ \begin{array}{cc} \nu_{k}^{j}\left(S^{j};\pi_{+}^{j}\right)=0 & k=1,\ldots ,m \end{array} \end{array}\right. \end{array}$$

where $\vartheta^{j}\left(S^{j};\pi_{+}^{j}\right)$, $\gamma_{i}^{j}\left(S^{j};\pi_{+}^{j}\right)\geq 0$ and $\nu_{k}^{j}\left(S^{j};\pi_{+}^{j}\right)=0$ are the objective function, inequality and equality constraints of $S^{j}$ given the predicted density $\pi_{+}^{j}(X)$.

The ultimate goal for a MTT network is to optimize the tracking precision. As is known to all, the MSE between target state and its estimate is currently the most widely-used evaluation indicator for tracking accuracy. Given the selected SN subset $S^{j}$ for the LFC j∈C, the MSE ${\left[\sigma_{S^{j}}^{j}\right]}^{2}$ between X and its Bayesian estimate ${\hat{X}}^{j}\left(Z^{S^{j}}\right)$ is

(16) $$\begin{array}{ll} {\left[\sigma_{S^{j}}^{j}\right]}^{2} & =E\left[e^{2}\left(X,{\hat{X}}^{j}\left(Z^{S^{j}}\right)\right)\right]\\ & =\int_{{\mathbb{Z}}^{S^{j}}}\int_{X\times L}f\left(X,Z^{S^{j}}\right)e^{2}\left(X,{\hat{X}}^{j}\left(Z^{S^{j}}\right)\right)\delta X\delta Z^{S^{j}}\\ & =\int_{{\mathbb{Z}}^{S^{j}}}\int_{X\times L}\prod_{s\in S^{j}}g^{s}\left(Z^{s}|X\right)\pi_{+}^{j}(X)e^{2}\left(X,{\hat{X}}^{j}\left(Z^{S^{j}}\right)\right)\delta X\delta Z^{S^{j}} \end{array}$$

where ${\mathbb{Z}}^{S^{j}}$ is the joint measurement space of the SN set $S^{j}$, $f\left(X,Z^{S^{j}}\right)$ is the joint density of $\left(X,Z^{S^{j}}\right)$, $e\left(X,{\hat{X}}^{j}\left(Z^{S^{j}}\right)\right)$ denotes the error distance between X and ${\hat{X}}^{j}\left(Z^{S^{j}}\right)$. In this paper, $e\left(X,{\hat{X}}^{j}\left(Z^{S^{j}}\right)\right)$ is measured by the 2nd-order LA metric ${\bar{d}}_{2}^{\left(c\right)}\left(X,{\hat{X}}^{j}\left(Z^{S^{j}}\right)\right)$ in (9).

Nevertheless, ${\left[\sigma_{S^{j}}^{j}\right]}^{2}$ cannot be used as the objective function of sensor selection optimization in (15) because ${\hat{X}}^{j}\left(Z^{S^{j}}\right)$ is unknown before sensor selection. To solve this, we replace ${\left[\sigma_{S^{j}}^{j}\right]}^{2}$ with its lower bound ${\left[{\underline{\sigma }}_{S^{j}}^{j}\right]}^{2}$, which provides an online indication on the limit of MTT accuracy within the labeled RFS framework.

Treating $\pi_{+}^{j}(X)$ as a default condition, (15) is finally rewritten as

(17) $$\begin{array}{l} {\left[S^{j}\right]}^{*}=\underset{S^{j}\subseteq N^{j}}{\arg\min}\;{\left[{\underline{\sigma }}_{S^{j}}^{j}\right]}^{2}\\ s.t.\left\{ \begin{array}{l} \begin{array}{cc} \gamma_{i}^{j}\left(S^{j}\right)\geq 0 & i=1,\ldots ,l \end{array}\\ \begin{array}{cc} \nu_{k}^{j}\left(S^{j}\right)=0 & k=1,\ldots ,m \end{array} \end{array}\right. \end{array}$$

## 4. Lower Bound For LA Metric Based MSE and Sub-Optimization For Sensor Selection

### 4.1. Derivation of LA Bound

In Section 4.1, Section 4.2, Section 4.3 and Appendix B, the superscript ‘j’ for the index of LFC is omitted. For example, ${\left({\underline{\sigma }}_{S^{j}}^{j}\right)}^{2}$ is abbreviated as ${\underline{\sigma }}_{S}^{2}$.

In order to derive ${\underline{\sigma }}_{S}^{2}$, it is assumed that

**A1:** Multi-target Bayesian recursion is a Mδ-GLMB RFS [22]. As a result, the predicted density $\pi_{+}(X)$ and posterior density $\pi \left(X|Z^{S}\right)$ can be described as $\pi_{+}={\left\{\left(\omega_{I,+},p_{I,+}\right)\right\}}_{I\in ℱ(L)}$ and $\pi \left(\cdot |Z^{S}\right)={\left\{\left(\omega_{I}\left(Z^{S}\right),p_{I}\left(\cdot |Z^{S}\right)\right)\right\}}_{I\in ℱ(L)}$.

**A2:** Although the optimal estimate of X can be extracted from the fused density $\bar{\pi }(X)$ by using the joint or marginal multi-target estimator [9], both the methods are very difficult to be implemented. Alternatively, the target number is firstly estimated according to the maximum a posterior (MAP) criterion and then the individual states are estimated according to the unbiased criterion under the obtained target number. In fact, the suboptimal method is applied in almost all multi-target Bayesian filters.

Let $Z_{m^{S}}^{S}=Z_{m^{s_{1}}}^{s_{1}},\ldots ,Z_{m^{s_{\left|S\right|}}}^{s_{\left|S\right|}}$ be the collection of measurement sets from the SN set S and ${\mathbb{Z}}_{m^{S}}^{S}={\mathbb{Z}}_{m^{s_{1}}}^{s_{1}}\times \cdots \times {\mathbb{Z}}_{m^{s_{\left|S\right|}}}^{s_{\left|S\right|}}$ be the space of $Z_{m^{S}}^{S}$, where $m^{s_{i}}$ is the number of measurements received by the SN $s_{i}$. Let $q\left(X_{n},Z_{m^{S}}^{S}\right)$ be the joint density over the space ${\left(X\times L\right)}_{n}\times {\mathbb{Z}}_{m^{S}}^{S}$. According to Bayesian formula, $q\left(X_{n},Z_{m^{S}}^{S}\right)$ is written as

(18) $$q\left(X_{n},Z_{m^{S}}^{S}\right)=\frac{1}{\Omega_{n,m^{S}}}\prod_{s\in S}g^{s}\left(Z_{m^{s}}^{s}|X_{n}\right)\pi_{+}\left(X_{n}\right)\;$$

where $\Omega_{n,m^{S}}$ is a normalization factor,

(19) $$\Omega_{n,m^{S}}=\sum_{\ell_{1:n}\in L_{n}}\int_{{\mathbb{Z}}_{m^{S}}^{S}}\int_{X_{n}}\prod_{s\in S}g^{s}\left(Z_{m^{s}}^{s}|X_{n}\right)\pi_{+}\left(X_{n}\right)dx_{1:n}dz_{1:m^{S}}^{S}\;$$

where $\int_{{\mathbb{Z}}_{m^{S}}^{S}}\begin{array}{c} \cdot \end{array}dz_{1:m^{S}}^{S}=\int_{{\mathbb{Z}}_{m^{s_{\left|S\right|}}}^{s_{\left|S\right|}}}\cdots \int_{{\mathbb{Z}}_{m^{s_{1}}}^{s_{1}}}\begin{array}{c} \cdot \end{array}dz_{1:m^{s_{1}}}^{s_{1}}\cdots dz_{1:m^{s_{\left|S\right|}}}^{s_{\left|S\right|}}$. (19) shows that $\Omega_{n,m^{S}}/\left(m^{S}!\cdot n!\right)$ is actually the probability $P\left(|X|=n,\left|Z^{S}\right|=m^{S}\right)$, where $m^{S}!=m^{s_{1}}!\cdots m^{s_{\left|S\right|}}!$ and $\left|Z^{S}\right|=m^{S}$ denotes $\left|Z^{s_{1}}\right|=m^{s_{1}},\ldots ,\left|Z^{s_{\left|S\right|}}\right|=m^{s_{\left|S\right|}}$. Substituting **A1** and (12) into (19) as well as using Lemma 12 in [17], $\Omega_{n,m^{S}}$ is obtained as

(20) $$\Omega_{n,m^{S}}=n!\left(\prod_{s\in S}e^{-\lambda^{s}}{\left[\lambda^{s}\right]}^{m^{s}}\right)\sum_{I\in {ℱ}_{n}(L)}\omega_{I,+}\sum_{\theta^{S}\in \Theta^{S}}\varphi_{I}\left(\theta^{S}\right)\;$$

where $\theta^{S}\in \Theta^{S}$ denotes $\theta^{s_{1}}\in \Theta^{s_{1}},\ldots ,\theta^{s_{\left|S\right|}}\in \Theta^{s_{\left|S\right|}}$,

(21) $$\varphi_{I}\left(\theta^{S}\right)={\langle p_{I,+}\left(\cdot ,\ell \right),\prod_{s\in S}\left(\delta_{0}\left(\theta^{s}(\ell )\right)\left(1-p_{d}^{s}\left(\cdot ,\ell \right)\right)+\left(1-\delta_{0}\left(\theta^{s}(\ell )\right)\right)\frac{p_{d}^{s}\left(\cdot ,\ell \right)}{\lambda^{s}}\right)\rangle }^{I}\;$$

Assume that $\theta^{s}(\ell )>0$ if s∈Y and $\theta^{s}(\ell )=0$ if s∈(S−Y). Then, (21) can be rewritten as

(22) $$\varphi_{I}\left(\theta^{S}\right)={\left(\sum_{Y\subseteq S}\frac{\langle p_{I,+}\left(\cdot ,\ell \right),p_{d}^{Y,S}\left(\cdot ,\ell \right)\rangle }{\prod_{s\in Y}\lambda^{s}}\right)}^{I}=\varphi_{I}(S)\;$$

where $\langle p_{I,+}\left(\cdot ,\ell \right),p_{d}^{Y,S}\left(\cdot ,\ell \right)\rangle =\int_{X_{1}}p_{I,+}\left(x,\ell \right)p_{d}^{Y,S}\left(x,\ell \right)dx$ denotes the inner product corresponding to x,

(23) $$p_{d}^{Y,S}(x)=\prod_{s\in Y}p_{d}^{s}(x)\prod_{s^{\prime }\in (S-Y)}\left(1-p_{d}^{s^{\prime }}(x)\right)\;$$

denotes the probability that only the SN subset Y⊆S receives the measurement from state x while the others miss the measurement.

(22) shows that $\varphi_{I}\left(\theta^{S}\right)$ is independent of the association mapping $\theta^{S}$. From (14), (20) and (22), $\Omega_{n,m^{S}}$ is finally obtained as

(24) $$\Omega_{n,m^{S}}=n!\left(\prod_{s\in S}e^{-\lambda^{s}}{\left[\lambda^{s}\right]}^{m^{s}}\chi_{n,m^{s}}\right)\sum_{I\in {ℱ}_{n}(L)}\omega_{I,+}\varphi_{I}(S)\;$$

Since $q\left(X_{n},Z_{m^{S}}^{S}\right)$ is permutation invariant over $x_{1:n}$, its marginal density over any of $x_{1:n}$ is the same and is obtained by

(25) $$q_{n}\left(x,Z_{m^{S}}^{S}\right)=\int_{X_{n-1}}q\left(\left\{x,x_{2:n}\right\},Z_{m^{S}}^{S}\right)dx_{2:n}\;$$

Substituting (18) into (25) as well as using **A1** and the identical equation $\delta_{n}\left(\left|\left\{\ell ,\ell_{2:n}\right\}\right|\right)=\delta_{n-1}\left(\left|\left\{\ell_{2:n}\right\}\right|\right)\left(1-1_{\left\{\ell_{2:n}\right\}}(\ell )\right)$, $q_{n}\left(x,Z_{m^{S}}^{S}\right)$ is written as

(26) $$\begin{array}{c} q_{n}\left(x,Z_{m^{S}}^{S}\right)=\frac{1}{\Omega_{n,m^{S}}}\sum_{\ell_{2:n}\in L_{n-1}}\delta_{n-1}\left(\left|\left\{\ell_{2:n}\right\}\right|\right)\left(1-1_{\left\{\ell_{2:n}\right\}}(\ell )\right)\sum_{I\in {ℱ}_{n}(L)}\omega_{I,+}\delta_{I}\left(\left\{\ell ,\ell_{2:n}\right\}\right)\\ \begin{array}{c} \cdot \end{array}\int_{X_{n-1}}\prod_{s\in S}g^{s}\left(Z_{m^{s}}^{s}|\left\{x,x_{2:n}\right\}\right)p_{I,+}(x)\prod_{t=2}^{n}p_{I,+}\left(x_{t}\right)dx_{2:n} \end{array}$$

Substituting (12) into (26) and then simplifying the result, we get

(27) $$q_{n}\left(x,Z_{m^{S}}^{S}\right)=\frac{1}{\Omega_{n,m^{S}}}\left(\prod_{s\in S}e^{-\lambda^{s}}{\left[\kappa^{s}\right]}^{Z_{m^{s}}^{s}}\right)\sum_{I\in {ℱ}_{n}(L)}\sum_{\theta^{S}\in \Theta^{S}}1_{I}(\ell )\omega_{I,+}\eta_{I,Z_{m^{S}}^{S}}\left(\ell ;\theta^{S}\right)q_{I}\left(x,Z_{m^{S}}^{S};\theta^{S}\right)\;$$

where $q_{I}\left(x,Z_{m^{S}}^{S};\theta^{S}\right)$ is the marginal density of $q\left(X,Z_{m^{S}}^{S}\right)$ over any of $x_{1:n}$ given the label set I and association mapping $\theta^{S}$,

(28) $$q_{I}\left(x,Z_{m^{S}}^{S};\theta^{S}\right)=\sum_{Y\subseteq S}p_{d}^{Y,S}(x)p_{I,+}(x)\prod_{s\in Y}g^{s}\left(z_{\theta^{s}(\ell )}^{s}|x\right)\;$$

(29) $$\eta_{I,Z_{m^{S}}^{S}}\left(\ell ,\theta^{S}\right)={\langle p_{I,+}\left(\cdot ,\ell^{\prime }\right),\prod_{s\in S}\psi_{Z_{m^{s}}^{s}}^{s}\left(\cdot ,\ell^{\prime };\theta^{s}-\theta^{s}(\ell )\right)\rangle }^{I-\ell }\;$$

where $\theta^{s}-\theta^{s}(\ell )$ denotes the remaining association mapping in $\theta^{s}$ except for $\theta^{s}(\ell )$.

MAP detection criterion determines $\left|\hat{X}\left(Z_{m^{S}}^{S}\right)\right|=\hat{n}$ ($\hat{n}=0,1,\ldots ,\infty$) if and only if $Z_{m^{S}}^{S}\subseteq {\mathbb{Z}}_{\hat{n},m^{S}}^{S}$,

(30) $${\mathbb{Z}}_{\hat{n},m^{S}}^{S}=\left\{\begin{array}{cc} Z_{m^{S}}^{S}\subseteq {\mathbb{Z}}_{m^{S}}^{S}: & \hat{n}=\underset{n}{\arg\max}\left(P\left(|X|=n|Z_{m^{S}}^{S}\right)\right) \end{array}\right\}\;$$

where ${\mathbb{Z}}_{\hat{n},m^{S}}^{S}={\mathbb{Z}}_{\hat{n},m^{s_{1}}}^{s_{1}}\times \cdots \times {\mathbb{Z}}_{\hat{n},m^{s_{\left|S\right|}}}^{s_{\left|S\right|}}$ is the joint measurement subspace of the SN set S where the target number is estimated as $\hat{n}$; ${\mathbb{Z}}_{0,m^{S}}^{S},{\mathbb{Z}}_{1,m^{S}}^{S},\ldots ,{\mathbb{Z}}_{\infty ,m^{S}}^{S}$ is a partition of ${\mathbb{Z}}_{m^{S}}^{S}$; $P\left(|X|=n|Z_{m^{S}}^{S}\right)$ is the posterior probability of |X|=n given $Z_{m^{S}}^{S}$. From (6), $P\left(|X|=n|Z_{m^{S}}^{S}\right)$ is written as

(31) $$P\left(|X|=n|Z_{m^{S}}^{S}\right)=\sum_{I\in {ℱ}_{n}(L)}\omega_{I}\left(Z_{m^{S}}^{S}\right)\;$$

where $\omega_{I}\left(Z_{m^{S}}^{S}\right)$ is the existing probability of the label set I given $Z_{m^{S}}^{S}$. According to the update step of Mδ-GLMB [22], $\omega_{I}\left(Z_{m^{S}}^{S}\right)$ is obtained as

(32) $$\omega_{I}\left(Z_{m^{S}}^{S}\right)=\sum_{\theta^{S}\in \Theta^{S}}\frac{\omega_{I,+}\beta_{I,Z_{m^{S}}^{S}}\left(\theta^{S}\right)}{\sum_{I^{\prime }\in ℱ(L)}\sum_{{\theta^{\prime }}^{S}\in \Theta^{S}}\omega_{I^{\prime },+}\beta_{I^{\prime },Z_{m^{S}}^{S}}\left({\theta^{\prime }}^{S}\right)}\;$$

(33) $$\beta_{I,Z_{m^{S}}^{S}}\left(\theta^{S}\right)={\langle p_{I,+}\left(\cdot ,\ell \right),\prod_{s\in S}\psi_{Z_{m^{s}}^{s}}^{s}\left(\cdot ,\ell ;\theta^{s}\right)\rangle }^{I}\;$$

where we have $\beta_{I,Z_{m^{S}}^{S}}\left(\theta^{S}\right)=\sum_{\ell \in L_{1}}\eta_{I,Z_{m^{S}}^{S}}\left(\ell ,\theta^{S}\right)$ from (29) and (33).

Let $\Psi_{\hat{n},n,m^{S}}$ be the integral of $q\left(X_{n},Z_{m^{S}}^{S}\right)$ over the space ${\left(X\times L\right)}_{n}\times {\mathbb{Z}}_{\hat{n},m^{S}}^{S}$. From (18), $\Psi_{\hat{n},n,m^{S}}$ can be written as

(34) $$\begin{array}{ll} \Psi_{\hat{n},n,m^{S}} & =\sum_{\ell_{1:n}\in L_{n}}\int_{{\mathbb{Z}}_{\hat{n},m^{S}}^{S}}\int_{X_{n}}q\left(X_{n},Z_{m^{S}}^{S}\right)dx_{1:n}dz_{1:m^{S}}^{S}\\ & =\frac{1}{\Omega_{n,m^{S}}}\sum_{\ell_{1:n}\in L_{n}}\int_{{\mathbb{Z}}_{\hat{n},m^{S}}^{S}}\int_{X_{n}}\prod_{s\in S}g^{s}\left(Z_{m^{s}}^{s}|X_{n}\right)\pi_{+}\left(X_{n}\right)dx_{1:n}dz_{1:m^{S}}^{S} \end{array}$$

(34) shows that $\Omega_{n,m^{S}}\Psi_{\hat{n},n,m^{S}}/\left(m^{S}!\cdot n!\right)$ is actually the probability $P\left(\left|\hat{X}\left(Z_{m^{S}}^{S}\right)\right|=\hat{n},|X|=n,\left|Z^{S}\right|=m^{S}\right)$. Substituting **A1** and (12) into (34) as well as using Lemma 12 in [17], $\Psi_{\hat{n},n,m^{S}}$ is obtained as

(35) $$\Psi_{\hat{n},n,m^{S}}=\frac{n!}{\Omega_{n,m^{S}}}\left(\prod_{s\in S}e^{-\lambda^{s}}{\left[\lambda_{\hat{n}}^{s}\right]}^{m^{s}}\right)\sum_{I\in {ℱ}_{n}(L)}\sum_{\theta^{S}\in \Theta^{S}}\omega_{I,+}\phi_{I,\hat{n}}\left(\theta^{S}\right)\;$$

where

(36) $$\lambda_{\hat{n}}^{s}=\int_{{\mathbb{Z}}_{\hat{n}，1}^{s}}\kappa^{s}\left(z^{s}\right)dz^{s}\;$$

(37) $$\phi_{I,\hat{n}}\left(\theta^{S}\right)={\langle p_{I,+}\left(\cdot ,\ell \right),\prod_{s\in S}\left(\delta_{0}\left(\theta^{s}(\ell )\right)\left(1-p_{d}^{s}\left(\cdot ,\ell \right)\right)+\left(1-\delta_{0}\left(\theta^{s}(\ell )\right)\right)\frac{p_{d}^{s}\left(\cdot ,\ell \right)\int_{{\mathbb{Z}}_{\hat{n}，1}^{s}}g^{s}\left(z^{s}|\cdot ,\ell \right)dz^{s}}{\lambda_{\hat{n}}^{s}}\right)\rangle }^{I}\;$$

Similar with $\varphi_{I}\left(\theta^{S}\right)$, $\phi_{I,\hat{n}}\left(\theta^{S}\right)$ can be rewritten as

(38) $$\phi_{I,\hat{n}}\left(\theta^{S}\right)={\left(\sum_{Y\subseteq S}\frac{\langle p_{I,+}\left(\cdot ,\ell \right)\prod_{s\in Y}\int_{{\mathbb{Z}}_{\hat{n}，1}^{s}}g^{s}\left(z^{s}|\cdot ,\ell \right)dz^{s},p_{d}^{Y,S}\left(\cdot ,\ell \right)\rangle }{\prod_{s\in Y}\lambda_{\hat{n}}^{s}}\right)}^{I}=\phi_{I,\hat{n}}(S)\;$$

(38) shows that $\phi_{I,\hat{n}}\left(\theta^{S}\right)$ is independent of the association mapping $\theta^{S}$. From (14), (35) and (38), $\Psi_{\hat{n},n,m^{S}}$ is finally obtained as

(39) $$\Psi_{\hat{n},n,m^{S}}=\frac{n!}{\Omega_{n,m^{S}}}\left(\prod_{s\in S}e^{-\lambda^{s}}{\left[\lambda_{\hat{n}}^{s}\right]}^{m^{s}}\chi_{n,m^{s}}\right)\sum_{I\in {ℱ}_{n}(L)}\omega_{I,+}\phi_{I,\hat{n}}(S)\;$$

***Theorem 1:*** Given **A1**, **A2** and the SN set S, the lower bound for the 2nd-order LA metric based MSE of (16) is

(40) $${\underline{\sigma }}_{S}^{2}=\sum_{m^{S}=0}^{\infty }\sum_{n=0}^{\infty }\sum_{\hat{n}=0,n+\hat{n}>0}^{\infty }\sum_{k=0}^{\min(n,\hat{n})}\sum_{\ell \in L_{1}}\frac{\Omega_{n,m^{S}}\Psi_{\hat{n},n,m^{S}}}{m^{S}!\left(n-k\right)!}\left(\varepsilon_{k,\hat{n},n}\min\left(c^{2},\frac{1}{\Psi_{\hat{n},n,m^{S}}}\sum_{l=1}^{L}{\left[J_{\hat{n},n,m^{S}}^{-1}(\ell )\right]}^{l,l}\right)+\left(1-\varepsilon_{k,\hat{n},n}\right)c^{2}\right)\;$$

where c is the cut-off of the LA metric, L is the dimension of x, $\Omega_{n,m^{S}}$ and $\Psi_{\hat{n},n,m^{S}}$ are given in (24) and (39),

(41) $$\varepsilon_{k,\hat{n},n}=\frac{k}{n+\hat{n}-k},\;k=0,1,\ldots ,\min\left(n,\hat{n}\right)\;$$

is actually the possible ratio of the number of common labels to the number of all distinct labels for the multi-target states and their estimates given $\left|\hat{X}\left(Z_{m^{S}}^{S}\right)\right|=\hat{n},|X|=n,\left|Z^{S}\right|=m^{S}$ (that is, $\left|ℒ\left(X_{n}\right)\cap ℒ\left({\hat{X}}_{\hat{n}}\left(Z_{m^{S}}^{S}\right)\right)\right|/\left|ℒ\left(X_{n}\right)\cup ℒ\left({\hat{X}}_{\hat{n}}\left(Z_{m^{S}}^{S}\right)\right)\right|$), $J_{\hat{n},n,m^{S}}(\ell )$ is the L×L FIM for a single-target state with the label ℓ given $\left|\hat{X}\left(Z_{m^{S}}^{S}\right)\right|=\hat{n},|X|=n,\left|Z^{S}\right|=m^{S}$,

(42) $${\left[J_{\hat{n},n,m^{S}}(\ell )\right]}^{i,j}=-\frac{1}{\Psi_{\hat{n},n,m^{S}}^{2}}\int_{{\mathbb{Z}}_{\hat{n},m^{S}}^{S}}\int_{X_{1}}\Phi_{n}\left(x,\ell ,Z_{m^{S}}^{S}\right)dxdz_{1:m^{S}}^{S}\begin{array}{c} \begin{array}{cc} & i,j=1,\ldots ,L \end{array} \end{array}\;$$

where the integral region ${\mathbb{Z}}_{\hat{n},m^{S}}^{S}$ for measurement is given by (30), $J_{\hat{n},n,m^{S}}(\ell )=\infty$ if ${\mathbb{Z}}_{\hat{n},m^{S}}^{S}=\emptyset$, the integrand $\Phi_{n}\left(x,\ell ,Z_{m^{S}}^{S}\right)$ is

(43) $$\begin{array}{ll} \Phi_{n}\left(x,\ell ,Z_{m^{S}}^{S}\right) & =q_{n}\left(x,\ell ,Z_{m^{S}}^{S}\right)\frac{\partial^{2}\log q_{n}\left(x,\ell ,Z_{m^{S}}^{S}\right)}{\partial x^{i}\partial x^{j}}\\ & =-\frac{1}{q_{n}\left(x,\ell ,Z_{m^{S}}^{S}\right)}\cdot \frac{\partial q_{n}\left(x,\ell ,Z_{m^{S}}^{S}\right)}{\partial x^{i}}\cdot \frac{\partial q_{n}\left(x,\ell ,Z_{m^{S}}^{S}\right)}{\partial x^{j}}+\frac{\partial^{2}q_{n}\left(x,\ell ,Z_{m^{S}}^{S}\right)}{\partial x^{i}\partial x^{j}} \end{array}$$

where $q_{n}\left(x,\ell ,Z_{m^{S}}^{S}\right)$ is given in (27).

See Appendix B for proof of Theorem 1.

**Remark** **2.** *The number of estimated targets is assumed to be unknown in the derivation of the proposed bound. Only the MAP rule, rather than the specific (or exact) number of estimated targets, is required to obtain the bound. The symbol $\hat{n}$ used for calculating the bound in Theorem 1 is just an index for all possible (or unknown) number of estimated targets. This is similar with the symbols ℓ, k, n and $m^{S}$ in Theorem 1, which are just the indices for the labels, the number of common labels in true targets and their estimates, the number of true targets and the number of sensor measurements, respectively.*

Furthermore, the reason for imposing the MAP rule has been explained in **A2**. (30) has also shown that the measurement space ${\mathbb{Z}}_{m^{S}}^{S}$ can be divided into ${\mathbb{Z}}_{0,m^{S}}^{S},{\mathbb{Z}}_{1,m^{S}}^{S},\ldots ,{\mathbb{Z}}_{\infty ,m^{S}}^{S}$ according to the MAP rule. It is very helpful for the proof of Theorem 1.

**Remark** **3.** *In general, the maximum number of targets and measurements can be presented by prior knowledge. Moreover, the label space $L_{1}={L^{\prime }}_{1}\cup B_{1}$, where ${L^{\prime }}_{1}$ and $B_{1}$ are the label spaces for the last time and new-born targets. In general, $B_{1}$ can also be preseted by prior knowledge. According to these presets, the sum of infinite terms in (40) becomes the sum of finite terms.*

**Remark** **4.** *Once the specific forms of $p_{I,+}(x)$, $p_{d}(x)$ and $g^{s}\left(z^{s}|x\right)$ are given, $\partial q_{n}\left(x,\ell ,Z_{m^{S}}^{S}\right)/\partial x^{i}$ and $\partial^{2}q_{n}\left(x,\ell ,Z_{m^{S}}^{S}\right)/\partial x^{i}\partial x^{j}$ in $\Phi_{n}\left(x,\ell ,Z_{m^{S}}^{S}\right)$ can be obtained from (27) and (28).*

**Remark** **5.** *The formulas of $\Psi_{\hat{n},n,m^{S}}$ and $J_{\hat{n},n,m^{S}}(\ell )$ contain the integral over the measurement subspace ${\mathbb{Z}}_{\hat{n},m^{S}}^{S}$, which is calculated via MC integration [45]. To improve computational efficiency, the samples of MC integration are selected as predicted ideal measurement sets (PIMS) [31]. The calculation steps are shown in Algorithm 2.*

**Algorithm 2.** Steps for calculating $\Psi_{\hat{n},n,m^{S}}$ and $J_{\hat{n},n,m^{S}}(\ell )$.**1. Prediction sampling:** Generate M samples ${\tilde{X}}_{n,+}^{\left(1\right)},\ldots ,{\tilde{X}}_{n,+}^{\left(M\right)}$ of multi-target state sets from the predicted density $\pi_{+}(X)$;**2. PIMS generating:** For j=1,…,M, generate PIMS ${\tilde{Z}}_{m^{S}}^{S,(j)}$ of the SN set S based on ${\tilde{X}}_{n,+}^{\left(j\right)}$ [31];**3. PIMS partitioning:** Divide PIMS ${\left\{{\tilde{Z}}_{m^{S}}^{S,(j)}\right\}}_{j=1}^{M}$ into the measurement subspace ${\mathbb{Z}}_{\hat{n},m^{S}}^{S}$ ($\hat{n}=0,1,\ldots ,\infty$) according to (30);**4. MC integration:** Given the PIMS assigned to ${\mathbb{Z}}_{\hat{n},m^{S}}^{S}$, $\Psi_{\hat{n},n,m^{S}}$ and $J_{\hat{n},n,m^{S}}(\ell )$ are obtained by applying MC integral formula [45] to (39) and (42).

### 4.2. SMC and GM Implementations for the Bound

In order to derive the SMC implementation of the bound in Theorem 1, it is assumed that

**A3:** Each $p_{I,+}(x)$ involved in the predicted Mδ-GLMB density $\pi_{+}={\left\{\left(\omega_{I,+},p_{I,+}\right)\right\}}_{I\in ℱ(L)}$ is described by a set of weighted particles ${\left\{\left({\tilde{\upsilon }}_{I,+}^{i}(\ell ),{\tilde{x}}_{I,+}^{i}(\ell )\right)\right\}}_{i=1}^{{\tilde{G}}_{I,+}(\ell )}$,

(44) $$p_{I,+}(x)=\sum_{i=1}^{{\tilde{G}}_{I,+}(\ell )}{\tilde{\upsilon }}_{I,+}^{i}(\ell )\delta_{{\tilde{x}}_{I,+}^{i}(\ell )}(x)\;$$

Substituting (44) into (22), (28), (29), (33) and (38), $\varphi_{I}(S)$, $q_{I}\left(x,Z_{m^{S}}^{S};\theta^{S}\right)$, $\eta_{I,Z_{m^{S}}^{S}}\left(\ell ,\theta^{S}\right)$, $\beta_{I,Z_{m^{S}}^{S}}\left(\theta^{S}\right)$ and $\phi_{I,\hat{n}}(S)$ are rewritten as

(45) $$\varphi_{I}(S)={\left(\sum_{Y\subseteq S}\frac{\sum_{i=1}^{{\tilde{G}}_{I,+}(\ell )}{\tilde{\upsilon }}_{I,+}^{i}(\ell )p_{d}^{Y,S}\left({\tilde{x}}_{I,+}^{i}(\ell ),\ell \right)}{\prod_{s\in Y}\lambda^{s}}\right)}^{I}\;$$

(46) $$q_{I}\left(x,Z_{m^{S}}^{S};\theta^{S}\right)=\sum_{Y\subseteq S}\sum_{i=1}^{{\tilde{G}}_{I,+}(\ell )}{\tilde{\upsilon }}_{I,+}^{i}(\ell )p_{d}^{Y,S}\left({\tilde{x}}_{I,+}^{i}(\ell ),\ell \right)\prod_{s\in Y}g^{s}\left(z_{\theta^{s}(\ell )}^{s}|{\tilde{x}}_{I,+}^{i}(\ell ),\ell \right)\;$$

(47) $$\eta_{I,Z_{m^{S}}^{S}}\left(\ell ,\theta^{S}\right)={\left(\sum_{i=1}^{{\tilde{G}}_{I,+}(\ell^{\prime })}{\tilde{\upsilon }}_{I,+}^{i}(\ell^{\prime })\prod_{s\in S}\psi_{Z_{m^{s}}^{s}}^{s}\left({\tilde{x}}_{I,+}^{i}(\ell^{\prime }),\ell^{\prime };\theta^{s}-\theta^{s}(\ell )\right)\right)}^{I-\ell }\;$$

(48) $$\beta_{I,Z_{m^{S}}^{S}}\left(\theta^{S}\right)={\left(\sum_{i=1}^{{\tilde{G}}_{I,+}(\ell )}{\tilde{\upsilon }}_{I,+}^{i}(\ell )\prod_{s\in S}\psi_{Z_{m^{s}}^{s}}^{s}\left({\tilde{x}}_{I,+}^{i}(\ell ),\ell ;\theta^{s}\right)\right)}^{I}\;$$

(49) $$\phi_{I,\hat{n}}(S)={\left(\sum_{Y\subseteq S}\frac{\sum_{i=1}^{{\tilde{G}}_{I,+}(\ell )}{\tilde{\upsilon }}_{I,+}^{i}(\ell )p_{d}^{Y,S}\left({\tilde{x}}_{I,+}^{i}(\ell ),\ell \right)\prod_{s\in Y}\int_{{\mathbb{Z}}_{\hat{n}，1}^{s}}g^{s}\left(z^{s}|{\tilde{x}}_{I,+}^{i}(\ell ),\ell \right)dz^{s}}{\prod_{s\in Y}\lambda_{\hat{n}}^{s}}\right)}^{I}\;$$

Finally, the SMC forms of $\Omega_{n,m^{S}}$, $q_{n}\left(x,Z_{m^{S}}^{S}\right)$, $\Psi_{\hat{n},n,m^{S}}$ and $\Phi_{n}\left(x,\ell ,Z_{m^{S}}^{S}\right)$ are respectively obtained by substituting (45)–(49) into (24), (27), (39) and (43), where $\partial q_{n}\left(x,\ell ,Z_{m^{S}}^{S}\right)/\partial x^{i}$ and $\partial^{2}q_{n}\left(x,\ell ,Z_{m^{S}}^{S}\right)/\partial x^{i}\partial x^{j}$ involved in $\Phi_{n}\left(x,\ell ,Z_{m^{S}}^{S}\right)$ are

(50) $$\left\{ \begin{array}{l} \frac{\partial q_{n}\left(x,\ell ,Z_{m^{S}}^{S}\right)}{\partial x^{i}}=\sum_{i^{\prime }=1}^{{\tilde{G}}_{I,+}(\ell )}{\tilde{\upsilon }}_{I,+}^{i^{\prime }}(\ell ){\frac{\partial q_{n}\left(x,\ell ,Z_{m^{S}}^{S}\right)}{\partial x^{i}}|}_{x={\tilde{x}}_{I,+}^{i^{\prime }}(\ell )}\\ \frac{\partial^{2}q_{n}\left(x,\ell ,Z_{m^{S}}^{S}\right)}{\partial x^{i}\partial x^{j}}=\sum_{i^{\prime }=1}^{{\tilde{G}}_{I,+}(\ell )}{\tilde{\upsilon }}_{I,+}^{i^{\prime }}(\ell ){\frac{\partial^{2}q_{n}\left(x,\ell ,Z_{m^{S}}^{S}\right)}{\partial x^{i}\partial x^{j}}|}_{x={\tilde{x}}_{I,+}^{i^{\prime }}(\ell )} \end{array}\right.\;$$

In order to derive the GM implementation of the bound in Theorem 1, it is assumed that

**A4:** Each $p_{I,+}(x)$ involved in the predicted Mδ-GLMB density $\pi_{+}={\left\{\left(\omega_{I,+},p_{I,+}\right)\right\}}_{I\in ℱ(L)}$ is described by the GM form of

(51) $$p_{I,+}(x)=\sum_{i=1}^{G_{I,+}(\ell )}\upsilon_{I,+}^{i}(\ell )N\left(x;\mu_{I,+}^{i}(\ell ),\Sigma_{I,+}^{i}(\ell )\right),\;\mathrm{with}\;\sum_{i=1}^{G_{I,+}(\ell )}\upsilon_{I,+}^{i}(\ell )=1\;$$

where $N\left(\cdot ;\mu_{I,+}^{i}(\ell ),\Sigma_{I,+}^{i}(\ell )\right)$ denotes the Gaussian density with mean $\mu_{I,+}^{i}(\ell )$ and covariance matrix $\Sigma_{I,+}^{i}(\ell )$, $\upsilon_{I,+}^{i}(\ell )$ and $G_{I,+}(\ell )$ are the weights and number of GM terms.

**A5:** The detection probability $p_{d}^{s}(x)$ is independent of x and the likelihood function $g^{s}\left(z^{s}|x\right)$ is linear Gaussian,

(52) $$p_{d}^{s}(x)=p_{d}^{s},\;g^{s}\left(z^{s}|x\right)=N\left(z^{s};H^{s}x,R^{s}\right)\;$$

where $H^{s}$ and $R^{s}$ are the observation function and covariance matrix for measurement noise.

From **A5** and (23), we have

(53) $$p_{d}^{Y,S}=\prod_{s\in Y}p_{d}^{s}\prod_{s^{\prime }\in (S-Y)}\left(1-p_{d}^{s^{\prime }}\right)\;$$

(54) $$\prod_{s\in Y}g^{s}\left(z^{s}|x\right)=N\left(z^{Y};H^{Y}x,R^{Y}\right)\;$$

where

(55) $$z^{Y}=\left[\begin{array}{c} z^{s_{1}}\\ \vdots \\ z^{s_{\left|Y\right|}} \end{array}\right];\;H^{Y}=\left[\begin{array}{c} H^{s_{1}}\\ \vdots \\ H^{s_{\left|Y\right|}} \end{array}\right];\;R^{Y}=\left[\begin{array}{ccc} R^{s_{1}} & & \\ & \ddots & \\ & & R^{s_{\left|Y\right|}} \end{array}\right]\;$$

Substituting (53) into (22), $\varphi_{I}(S)$ is rewritten as

(56) $$\varphi_{I}(S)={\left(\sum_{Y\subseteq S}\frac{p_{d}^{Y,S}}{\prod_{s\in Y}\lambda^{s}}\right)}^{\left|I\right|}\;$$

Substituting (51), (53) and (54) into (28) as well as using Lemma 2 in [11], $q_{I}\left(x,Z_{m^{S}}^{S};\theta^{S}\right)$ is rewritten as

(57) $$\begin{array}{ll} q_{I}\left(x,Z_{m^{S}}^{S};\theta^{S}\right) & =\sum_{Y\subseteq S}\sum_{i=1}^{G_{I,+}(\ell )}p_{d}^{Y,S}\upsilon_{I,+}^{i}(\ell )N\left(x;\mu_{I,+}^{i}(\ell ),\Sigma_{I,+}^{i}(\ell )\right)N\left(z_{\theta^{Y}(\ell )}^{Y};H^{Y}x,R^{Y}\right)\\ & =\sum_{Y\subseteq S}\sum_{i=1}^{G_{I,+}(\ell )}p_{d}^{Y,S}\upsilon_{I,+}^{i}(\ell )N\left(z_{\theta^{Y}(\ell )}^{Y};H^{Y}\mu_{I,+}^{i}(\ell ),\Xi_{I,+}^{i,Y}(\ell )\right)N\left(x;\mu_{I,Z_{m^{Y}}^{Y}}^{i}\left(\ell ;\theta^{Y}\right),\Sigma_{I}^{i}\left(\ell ;Y\right)\right) \end{array}$$

where

(58) $$\left\{ \begin{array}{l} \Xi_{I,+}^{i,Y}(\ell )=H^{Y}\Sigma_{I,+}^{i}(\ell ){\left[H^{Y}\right]}^{T}+R^{Y}\\ \mu_{I,Z_{m^{Y}}^{Y}}^{i}\left(\ell ;\theta^{Y}\right)=\mu_{I,+}^{i}(\ell )+\Sigma_{I,+}^{i}(\ell ){\left[H^{Y}\right]}^{T}{\left[\Xi_{I,+}^{i,Y}(\ell )\right]}^{-1}\left(z_{\theta^{Y}(\ell )}^{Y}-H^{Y}\mu_{I,+}^{i}(\ell )\right)\\ \Sigma_{I}^{i}\left(\ell ;Y\right)=\Sigma_{I,+}^{i}(\ell )-\Sigma_{I,+}^{i}(\ell ){\left[H^{Y}\right]}^{T}{\left[\Xi_{I,+}^{i,Y}(\ell )\right]}^{-1}H^{Y}\Sigma_{I,+}^{i}(\ell ) \end{array}\right.\;$$

Similarly, substituting (51), (53) and (54) into (29), (33) and (38), $\eta_{I,Z_{m^{S}}^{S}}\left(\ell ;\theta^{S}\right)$, $\beta_{I,Z_{m^{S}}^{S}}\left(\theta^{S}\right)$ and $\phi_{I,\hat{n}}(S)$ are rewritten as

(59) $$\eta_{I,Z_{m^{S}}^{S}}\left(\ell ;\theta^{S}\right)={\left(\sum_{Y\subseteq S}\sum_{i=1}^{G_{I,+}(\ell^{\prime })}\frac{p_{d}^{Y,S}\upsilon_{I,+}^{i}(\ell )}{\prod_{s\in Y}\kappa^{s}\left(z_{\left[\theta^{s}-\theta^{s}(\ell )\right](\ell^{\prime })}^{s}\right)}N\left(z_{\left[\theta^{Y}-\theta^{Y}(\ell )\right](\ell^{\prime })}^{Y};H^{Y}\mu_{I,+}^{i}(\ell ),\Xi_{I,+}^{i,Y}(\ell )\right)\right)}^{I-\ell }\;$$

(60) $$\beta_{I,Z_{m^{S}}^{S}}\left(\theta^{S}\right)={\left(\sum_{Y\subseteq S}\sum_{i=1}^{G_{I,+}(\ell )}\frac{p_{d}^{Y,S}\upsilon_{I,+}^{i}(\ell )}{\prod_{s\in Y}\kappa^{s}\left(z_{\theta^{s}(\ell )}^{s}\right)}N\left(z_{\theta^{Y}(\ell )}^{Y};H^{Y}\mu_{I,+}^{i}(\ell ),\Xi_{I,+}^{i,Y}(\ell )\right)\right)}^{I}\;$$

(61) $$\phi_{I,\hat{n}}(S)={\left(\sum_{Y\subseteq S}\sum_{i=1}^{G_{I,+}(\ell )}\frac{p_{d}^{Y,S}\upsilon_{I,+}^{i}(\ell )}{\prod_{s\in Y}\lambda_{\hat{n}}^{s}}\int_{{\mathbb{Z}}_{\hat{n}，1}^{Y}}N\left(z^{Y};H^{Y}\mu_{I,+}^{i}(\ell ),\Xi_{I,+}^{i,Y}(\ell )\right)dz^{Y}\right)}^{I}\;$$

Finally, the GM forms of $\Omega_{n,m^{S}}$, $q_{n}\left(x,Z_{m^{S}}^{S}\right)$, $\Psi_{\hat{n},n,m^{S}}$ and $\Phi_{n}\left(x,\ell ,Z_{m^{S}}^{S}\right)$ are respectively obtained by substituting (56)–(61) into (24), (27), (39) and (43). Obviously, they no longer contain integrals of state x and have analytic forms except for $\Psi_{\hat{n},n,m^{S}}$. Here $\partial q_{n}\left(x,\ell ,Z_{m^{S}}^{S}\right)/\partial x^{i}$ and $\partial^{2}q_{n}\left(x,\ell ,Z_{m^{S}}^{S}\right)/\partial x^{i}\partial x^{j}$ in $\Phi_{n}\left(x,\ell ,Z_{m^{S}}^{S}\right)$ are both linear functions of $\partial N\left(x;\mu_{I,Z_{m^{Y}}^{Y}}^{i}\left(\ell ;\theta^{Y}\right),\Sigma_{I}^{i}\left(\ell ;Y\right)\right)/\partial x^{i}$ and $\partial^{2}N\left(x;\mu_{I,Z_{m^{Y}}^{Y}}^{i}\left(\ell ;\theta^{Y}\right),\Sigma_{I}^{i}\left(\ell ;Y\right)\right)/\partial x^{i}\partial x^{j}$, which can be obtained by the following formulas:

(62) $$\left\{ \begin{array}{l} \frac{\partial N\left(x;\mu ,\Sigma \right)}{\partial x^{i}}=-{\left[\Sigma^{-1}\left(x-\mu \right)\right]}^{i}N\left(x;\mu ,\Sigma \right)\\ \frac{\partial^{2}N\left(x;\mu ,\Sigma \right)}{\partial x^{i}\partial x^{j}}={\left[\Sigma^{-1}\left(x-\mu \right){\left(x-\mu \right)}^{T}{\left[\Sigma^{-1}\right]}^{T}-\Sigma^{-1}\right]}^{i,j}N\left(x;\mu ,\Sigma \right) \end{array}\right.\;$$

If the observation model is non-linear, that is,

(63) $$g^{s}\left(z^{s}|x\right)=N\left(z^{s};h^{s}(x),R^{s}\right)\;$$

where $h^{s}(x)$ is the nonlinear observation function of state x. In this case, the extended Kalman (EK) or unscented Kalman (UK) filter can be used to calculate the mean and covariance matrix of each GM term [46,47].

### 4.3. Sub-Optimization Based on Coordinate Descent

The computational cost of this method is composed of three parts: sensor selection, Mδ-GLMB filtering and weighted KLA fusion. The latter two have been analyzed in Reference [22,23]. This paper only studies the computational cost of sensor selection and its approximate algorithm. When the number of SNs is large, it has a much more significant effect on the total amount of computation than the last two.

As shown in (15) and (17), sensor selection is actually a constrained combinatorial optimization problem. To find the optimal solution by the exhaustive search method, the objective function needs to be repeatedly calculated for $C_{\left|N\right|}^{\left|S\right|}=|N|!/\left(|S|!\left(\left|N|-|S\right|\right)!\right)$ times. Obviously, its computational cost increases with the SN number |N| in the form of combination explosion. In order to reduce the computational cost, some heuristic optimization algorithms, such as genetic algorithm [39] and so forth, is used to tackle this problem. However, the convergence speed of the heuristic algorithms will become rather show when the objective function is relatively complex. As a result, to further improve the computational speed, coordinate descent method [37] is proposed to find a sub-optimal solution of (17). Its computational cost increases with the SN number |N| in an approximate polynomial form.

Set a binary switch variable $\varsigma^{s}\in \{0,1\}$, s=1,…,|N|, for each SN. $\varsigma^{s}=1$ indicates s∈S while $\varsigma^{s}=0$ indicates s∉S. The vector $\varsigma =\left[\varsigma^{1},\ldots ,\varsigma^{\left|N\right|}\right]$ is composed of the switch variables of all SNs belonging to the same LFC. Clearly, the set S is completely determined by ς. Then, (17) is relaxed to an unconstrained optimization problem by the augmented objective function

(64) $$F\left(\varsigma ,r\right)={\underline{\sigma }}_{\varsigma }^{2}+\varpi \sum_{i=1}^{l}\gamma_{i}^{-1}(\varsigma )+\frac{1}{\sqrt{\varpi }}\sum_{j=1}^{m}\nu_{j}^{2}(\varsigma )\;$$

where ϖ>0 is a barrier factor, $\sum_{i=1}^{l}\gamma_{i}^{-1}(\varsigma )$ and $\sum_{j=1}^{m}\nu_{j}^{2}(\varsigma )$ are inequality and equality penalty terms.

Algorithm 3 presents the iteration steps for handling the relaxed problem by using the coordinate descent method.

**Algorithm 3.** Coordinate descent method.**Step 1:** Set initial iteration number i=0, initial SN switch vector $\varsigma_{\left(0\right)}$, initial barrier factor ϖ and its reduction coefficient 0<C<1;**Step 2:** From s=1 to s=|N|, calculate $\varsigma_{\left(i+1\right)}^{s}=\underset{\varsigma^{s}\in \{0,1\}}{\arg\min}F\left(\varsigma_{\left(i+1\right)}^{1},\ldots ,\varsigma_{\left(i+1\right)}^{s-1},\varsigma^{s},\varsigma_{\left(i\right)}^{s+1},\ldots ,\varsigma_{\left(i\right)}^{\left|N\right|},r\right)$, where $\varsigma_{\left(i+1\right)}^{1},\ldots ,\varsigma_{\left(i+1\right)}^{s-1},\varsigma_{\left(i\right)}^{s+1},\ldots ,\varsigma_{\left(i\right)}^{\left|N\right|}$ are treated as constants;**Step 3:** If $\varsigma_{\left(i+1\right)}=\varsigma_{\left(i\right)}$, then go to Step 4; Otherwise, set i=i+1, go to Step 2;**Step 4:** If $\varsigma_{\left(i+1\right)}=\varsigma_{\left(0\right)}$, then output $\varsigma_{\left(i+1\right)}$ as the solution of (17); Otherwise, set ϖ=Cϖ, $\varsigma_{\left(0\right)}=\varsigma_{\left(i+1\right)}$, i=0 and then go to Step 2.

In order to improve the probability to converge to the global optimum and accelerate the convergence speed for the coordinate descent method, the initial barrier factor ϖ and its reduction coefficient C can be appropriately selected by the methods in Reference [48], the initial SN switch vector is set as $\varsigma_{\left(0\right)}={\varsigma^{\prime }}^{*}$, where ${\varsigma^{\prime }}^{*}$ is the outputted switch vector at the last time.

### 4.4. Weighted KLA Fusion

**A1** indicates that the posterior density of each LFC is a Mδ-GLMB form of $\pi^{i}\left(X|Z^{S^{i}}\right)={\left\{\left(\omega_{I}^{i}\left(Z^{S^{i}}\right),p_{I}^{i}\left(x,\ell |Z^{S^{i}}\right)\right)\right\}}_{I\in ℱ(L)}$, i∈C. Then, for the LFC j∈C, given the LFC subset $C^{j}$ connected to it and the normalized nonnegative fusion weights $\alpha^{j,i}$ ($i\in C^{j}$), its fused density obtained by the weighted KLA rule is still a Mδ-GLMB form of ${\bar{\pi }}^{j}(X)={\left\{\left({\bar{\omega }}_{L}^{j},{\bar{p}}_{L}^{j}\left(x,\ell \right)\right)\right\}}_{L\in ℱ(L)}$ [23],

(65) $${\bar{\omega }}_{L}^{j}=\frac{\prod_{i\in C^{j}}{\left(\omega_{L}^{i}\left(Z^{S^{i}}\right)\right)}^{\alpha^{j,i}}{\left[\int \prod_{i\in C^{j}}{\left(p_{L}^{i}\left(x,\cdot |Z^{S^{i}}\right)\right)}^{\alpha^{j,i}}dx\right]}^{L}}{\sum_{L^{\prime }\subseteq L}\prod_{i^{\prime }\in C^{j}}{\left(\omega_{L^{\prime }}^{i^{\prime }}\left(Z^{S^{i^{\prime }}}\right)\right)}^{\alpha^{j,i^{\prime }}}{\left[\int \prod_{i^{\prime }\in C^{j}}{\left(p_{L^{\prime }}^{i^{\prime }}\left(x,\cdot |Z^{S^{i^{\prime }}}\right)\right)}^{\alpha^{j,i^{\prime }}}dx\right]}^{L^{\prime }}}\;$$

(66) $${\bar{p}}_{L}^{j}\left(x,\ell \right)=\frac{\prod_{i\in C^{j}}{\left(p_{L}^{i}\left(x,\ell |Z^{S^{i}}\right)\right)}^{\alpha^{j,i}}}{\int \prod_{i^{\prime }\in C^{j}}{\left(p_{L}^{i^{\prime }}\left(x,\ell |Z^{S^{i^{\prime }}}\right)\right)}^{\alpha^{j,i^{\prime }}}dx}\;$$

where the weight $\alpha^{j,i}$ reflects the effect of local posterior density $\pi^{i}\left(X|Z^{S^{i}}\right)$ on the fusion of the LFC j. The larger the weight is, the greater the impact it has on the KLA fusion.

The bound in Theorem 1 reflects the optimal MTT accuracy that is potentially achieved by a LFC after sensor selection. The larger the proposed bound of a LFC is, the worse the precision limit that it can achieve is. Therefore, the normalized weight $\alpha^{j,i}$ in the KLA fusion of the LFC j should be set inversely proportional to the proposed bound ${\left({\underline{\sigma }}_{S^{i}}^{i}\right)}^{2}$,

(67) $$\begin{array}{ccc} \alpha^{j,i}=\frac{{\left({\underline{\sigma }}_{S^{i}}^{i}\right)}^{-2}}{\sum_{i^{\prime }\in C^{j}}{\left({\underline{\sigma }}_{S^{i^{\prime }}}^{i^{\prime }}\right)}^{-2}}, & & i\in C^{j} \end{array}\;$$

which indicates that the larger the proposed bound of the LFC is, the smaller the proportion of its posterior density in the KLA fusion should be; and vice versa.

## 5. Simulations

The main goal of the simulations is to verify the following two points under different SNR conditions. First, our method conducts the sensor selection more effectively than the CS divergence based methods for the decentralized large-scale MTT network. This case is much more obvious when the sensors have different observation performance. Second, the coordinate descent method significantly shortens the calculation time of genetic algorithm at the expense of slight loss in tracking accuracy. To highlight these, the specific scenarios, including the multi-target dynamic model, sensor network architecture, observation model for SNs and so forth, are designed as follows.

Multiple targets move in a constant velocity (CV) model [49] over a two-dimensional region $A=[0,50]\times [0,50]{\mathrm{km}}^{2}$ and the number of targets is unknown and changes over time. The label of state x is noted as $\ell =\left(k^{b},i^{b}\right)$, where $k^{b}$ is the birth time and $i^{b}$ is the index to distinguish the birth targets at the same time. The unlabeled state is noted as $x={\left[p_{x},{\dot{p}}_{x},p_{y},{\dot{p}}_{y}\right]}^{T}$, where $\left(p_{x},p_{y}\right)$ and $\left({\dot{p}}_{x},{\dot{p}}_{y}\right)$ are the positions and velocities in the X and Y directions. The single-target transition density is in the Gaussian form of

(68) $$f\left(x,\ell |x^{\prime },\ell^{\prime }\right)=N\left(x;F_{\mathrm{CV}}x^{\prime },Q\right)\delta_{\ell^{\prime }}(\ell )\;$$

where $F_{\mathrm{CV}}$ and Q are the transition matrix and process noise covariance matrix for unlabeled state,

(69) $$F_{\mathrm{CV}}=\left[\begin{array}{cccc} 1 & \Delta & & \\ & 1 & & \\ & & 1 & \Delta \\ & & & 1 \end{array}\right],\;Q=q_{Q}^{2}\left[\begin{array}{cccc} \frac{\Delta^{4}}{4} & \frac{\Delta^{3}}{2} & & \\ \frac{\Delta^{3}}{2} & \Delta^{2} & & \\ & & \frac{\Delta^{4}}{4} & \frac{\Delta^{3}}{2}\\ & & \frac{\Delta^{3}}{2} & \Delta^{2} \end{array}\right]\;$$

where Δ is the sampling interval, $q_{Q}$ is the process noise standard deviation. In this example, Δ=10 s, $q_{Q}=0.002\;{\mathrm{km}/s}^{2}$ and survival probability $p_{s}(x)=0.95$.

The target birth is modeled as an LMB RFS with density $\pi^{b}={\left(\omega_{\ell }^{b},p_{\ell }^{b}\right)}_{\ell \in \{1,\ldots ,12\}}$, where $\omega_{\ell }^{b}$ and $p_{\ell }^{b}(x)$ are the existing probability and density of the new-birth target with label ℓ,

(70) $$p_{\ell }^{b}(x)=N\left(x;{\bar{x}}_{\ell }^{b},Q^{b}\right)\;$$

where in this example, $\omega_{\ell }^{b}=0.05$, $Q^{b}=\mathrm{diag}\left(25,10^{-2},25,10^{-2}\right)$ and ${\bar{x}}_{1}^{b}$~${\bar{x}}_{12}^{b}$ are ${\left[10,0.15,40,-0.15\right]}^{T}$, ${\left[20,0.1,40,-0.15\right]}^{T}$, ${\left[30,-0.1,40,-0.15\right]}^{T}$, ${\left[40,-0.15,40,-0.15\right]}^{T}$, ${\left[40,-0.15,30,-0.1\right]}^{T}$, ${\left[40,-0.15,20,0.1\right]}^{T}$, ${\left[40,-0.15,10,0.15\right]}^{T}$, ${\left[30,-0.1,10,0.15\right]}^{T}$, ${\left[20,0.1,10,0.15\right]}^{T}$, ${\left[10,0.15,10,0.15\right]}^{T}$, ${\left[10,0.15,20,0.1\right]}^{T}$, ${\left[10,0.15,30,-0.1\right]}^{T}$ in turn, where the units of position and speed are km and km/s.

**Figure 2:**
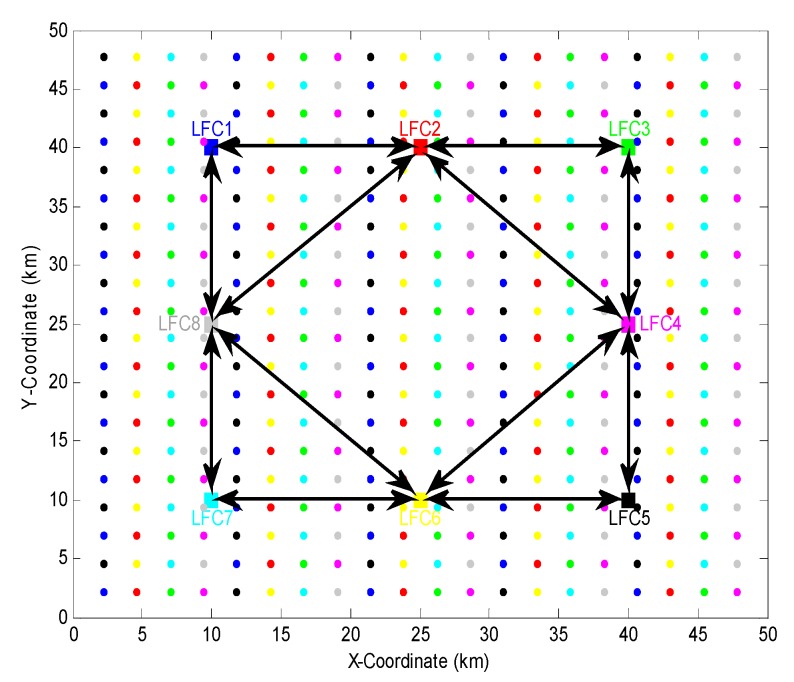
Locations of LFCs and SNs in the decentralized sensor network. ■ denotes LFC, ● denotes SN, different colors correspond to different LFCs and their subordinate SNs. Solid line with directed arrow indicates a communication connection between two LFCs. Each SN is 2.4 km apart away from other one.

The decentralized sensor network is composed of |C|=8 LFCs, which are noted as LFC1~LFC8. The positions (unit: km) of the LFCs are ${\left[10,40\right]}^{T}$, ${\left[25,40\right]}^{T}$, ${\left[40,40\right]}^{T}$, ${\left[40,25\right]}^{T}$, ${\left[40,10\right]}^{T}$, ${\left[25,10\right]}^{T}$, ${\left[10,10\right]}^{T}$, ${\left[10,25\right]}^{T}$ in turn. Each LFC has $\left|N^{j}\right|=50$ subordinate SNs (j=1,…,8). So there are in total |N|=… SNs in the entire network. Each LFC can communicate with other LFCs within 25 km apart away from it. Finally, the directed connection set A for the LFCs and the locations for all SNs are shown in Figure 2.

The clutter is a uniformly distributed Poisson RFS over the region A. In this example, the clutter rate and detection probability of each SN are firstly set as $\lambda^{s}=\lambda =20$ and $p_{d}^{s}(x)=p_{d}=0.95$, s=1,…,400.

To embody the performance variation of sensor observation, the network is assumed to consist of different types of SNs with distinctive observation function and noise covariance. The single-target likelihoods of the SNs are all Gaussian distributed as shown in (63). Each SN of LFC1 and LFC5 receives the distance and angle measurements of target, so its observation function $h^{s}(x)$ is

(71) $$h^{s}(x)={\left[\left\|x-u^{s}\right\|,\arctan\frac{p_{y}-u_{y}^{s}}{p_{x}-u_{x}^{s}}\right]}^{T},\;s\in N^{1}\;\mathrm{or}\;s\in N^{5}\;$$

where $u^{s}={\left[u_{x}^{s},u_{y}^{s}\right]}^{T}$ is the known position of the SN s, $\left\|x-u^{s}\right\|=\sqrt{{\left(p_{x}-u_{x}^{s}\right)}^{2}+{\left(p_{y}-u_{y}^{s}\right)}^{2}}$ is the distance between the SN s and target. The measurement noise covariance matrix is also modeled as a nonlinear function of state x,

(72) $$R^{s}(x)=\left\{ \begin{array}{l} \begin{array}{cc} \mathrm{diag}\left({\left(\left[0.2+0.05\left\|x-u^{s}\right\|\right]\mathrm{km}\right)}^{2},{\left(\left[0.02+0.001\left\|x-u^{s}\right\|\right]\mathrm{rad}\right)}^{2}\right) & s\in N^{1} \end{array}\\ \begin{array}{cc} \mathrm{diag}\left({\left(\left[0.4+0.04\left\|x-u^{s}\right\|\right]\mathrm{km}\right)}^{2},{\left(\left[0.04+0.0005\left\|x-u^{s}\right\|\right]\mathrm{rad}\right)}^{2}\right) & s\in N^{5} \end{array} \end{array}\right.\;$$

Each SN of LFC2 and LFC6 only receives the distance measurement of target. Its $h^{s}(x)$ and $R^{s}(x)$ are

(73) $$h^{s}(x)=\left\|x-u^{s}\right\|,\;s\in N^{2}\;\mathrm{or}\;s\in N^{6}\;$$

(74) $$R^{s}(x)=\left\{ \begin{array}{l} \begin{array}{cc} {\left(\left[0.1+0.02\left\|x-u^{s}\right\|\right]\mathrm{km}\right)}^{2} & s\in N^{2} \end{array}\\ \begin{array}{cc} {\left(\left[0.2+0.01\left\|x-u^{s}\right\|\right]\mathrm{km}\right)}^{2} & s\in N^{6} \end{array} \end{array}\right.\;$$

Each SN of LFC3 and LFC7 only receives the angle measurement of target. Its $h^{s}(x)$ and $R^{s}(x)$ are

(75) $$h^{s}(x)=\arctan\frac{p_{y}-u_{y}^{s}}{p_{x}-u_{x}^{s}},\;s\in N^{3}\;\mathrm{or}\;s\in N^{7}\;$$

(76) $$R^{s}(x)=\left\{ \begin{array}{l} \begin{array}{cc} {\left(\left[0.01+0.001\left\|x-u^{s}\right\|\right]\mathrm{rad}\right)}^{2} & s\in N^{3} \end{array}\\ \begin{array}{cc} {\left(\left[0.02+0.0005\left\|x-u^{s}\right\|\right]\mathrm{rad}\right)}^{2} & s\in N^{7} \end{array} \end{array}\right.\;$$

Each SN of LFC4 and LFC8 receives distance and Doppler measurements of target. Its $h^{s}(x)$ and $R^{s}(x)$ are

(77) $$h^{s}(x)={\left[\left\|x-u^{s}\right\|,\frac{\left(p_{x}-u_{x}^{s}\right){\dot{p}}_{x}+\left(p_{y}-u_{y}^{s}\right){\dot{p}}_{y}}{\left\|x-u^{s}\right\|}\right]}^{T},\;s\in N^{4}\;\mathrm{or}\;s\in N^{8}\;$$

(78) $$R^{s}(x)=\left\{ \begin{array}{l} \begin{array}{cc} \mathrm{diag}\left({\left(\left[0.2+0.05\left\|x-u^{s}\right\|\right]\mathrm{km}\right)}^{2},{\left(\left[0.02+0.001\left\|x-u^{s}\right\|\right]\mathrm{km}/s\right)}^{2}\right) & s\in N^{4} \end{array}\\ \begin{array}{cc} \mathrm{diag}\left({\left(\left[0.4+0.04\left\|x-u^{s}\right\|\right]\mathrm{km}\right)}^{2},{\left(\left[0.04+0.0005\left\|x-u^{s}\right\|\right]\mathrm{km}/s\right)}^{2}\right) & s\in N^{8} \end{array} \end{array}\right.\;$$

In this example, there are three constraints for the sensor selection optimization of (17), which are

**C1:** Due to the limitations of communication bandwidth, energy consumption, computation capacity and storage space, the LFC j can only select $K^{j}$ SNs at each scan ($K^{j}\leq \left|N^{j}\right|$),

(79) $$K^{j}-\left|S^{j}\right|=0\;$$

In this example, if j=1,4,5,8, then $K^{j}=5$; otherwise $K^{j}=8$.

**C2:** The field of view (FoV) of the SN s is modeled as a circular area with the center $u^{s}$ and radius $\rho^{s}$, $A^{s}\left(\rho^{s}\right)=\left\{\begin{array}{cc} p^{s}\in A: & \left\|p^{s}-u^{s}\right\|\leq \rho^{s} \end{array}\right\}$, $A^{s}\subseteq A$. The FoVs of different SNs can be overlapped. To ensure that the FoVs of the SN set $S^{j}$ can totally cover the region A, it is required that

(80) $$A-\underset{s\in S^{j}}{\cup }A^{s}\left(\rho^{s}\right)=0\;$$

In this example, if j=1,4,5,8, then $\rho^{s}=30\;\mathrm{km}$; otherwise $\rho^{s}=20\;\mathrm{km}$.

**C3:** To avoid mutual interference between the homogeneous SNs belonging to the same LFC, the distance between any two SNs in $S^{j}$ must be not smaller than the threshold $D^{j}$,

(81) $$\underset{s,s^{\prime }\in S^{j}}{\min}\left\|u^{s}-u^{s^{\prime }}\right\|-D^{j}\geq 0\;$$

In this example, $D^{j}=5\;\mathrm{km}$ for j=1,…,8.

According to the objective function and calculation method used for sensor selection, our algorithm is abbreviated as ***LA bound with coordinate descent***. It is firstly implemented by the SMC technique and coded by MATLAB R2018a. Each $p_{I}\left(\cdot ,\ell \right)$ involved in the Mδ-GLMB density is approximated by 500 particles on average. In this example, the maximum numbers of targets and measurements of each SN per scan are set as 25 and 200, the cut-off is set as c=1000 m. The algorithm is testing on a desktop with the CPU of AMD Ryzen 7 2700X and 64 GB RAM. We conduct 500 MC simulations, each of which includes T=25 scans (a total of 250 s). In these simulations, the target tracks (including the instants of birth and death), clutter and measurements originating from targets are generated independently according to the aforementioned models.

**Figure 3:**
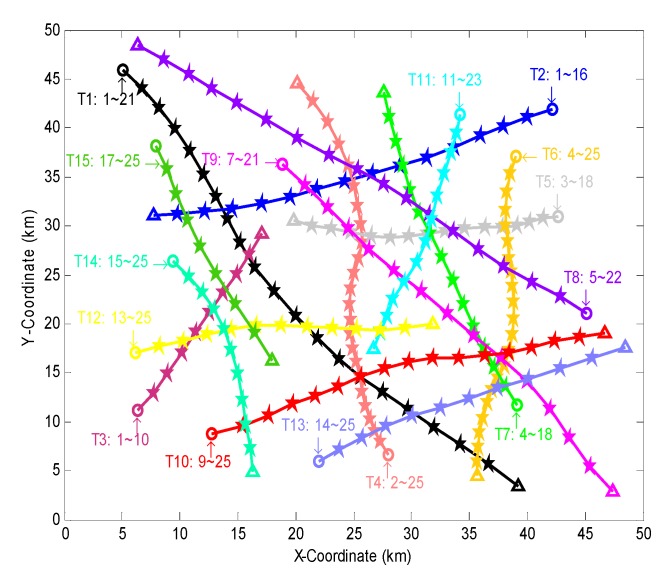
Target trajectories in a simulation. ○, △ and ★ are the start point, end point and rest positions of a target, the solid line is the track of a target, different colors correspond to different targets.

We firstly present the result of sensor selection obtained by the algorithm in one simulation. Figure 3 shows the target trajectories in the simulation, where a total of 15 targets are generated at different instants and locations. For easy description, the targets are named as T1~T15 in turn. The name and survival period of each target are marked at the start point of the target. During the surveillance period, the number of targets at the initial time is the least (3 targets). The number of targets at the 15th~18th scans is the most (13 targets). The target T1 intersects with the target T10 at the 15th scan.

**Figure 4:**
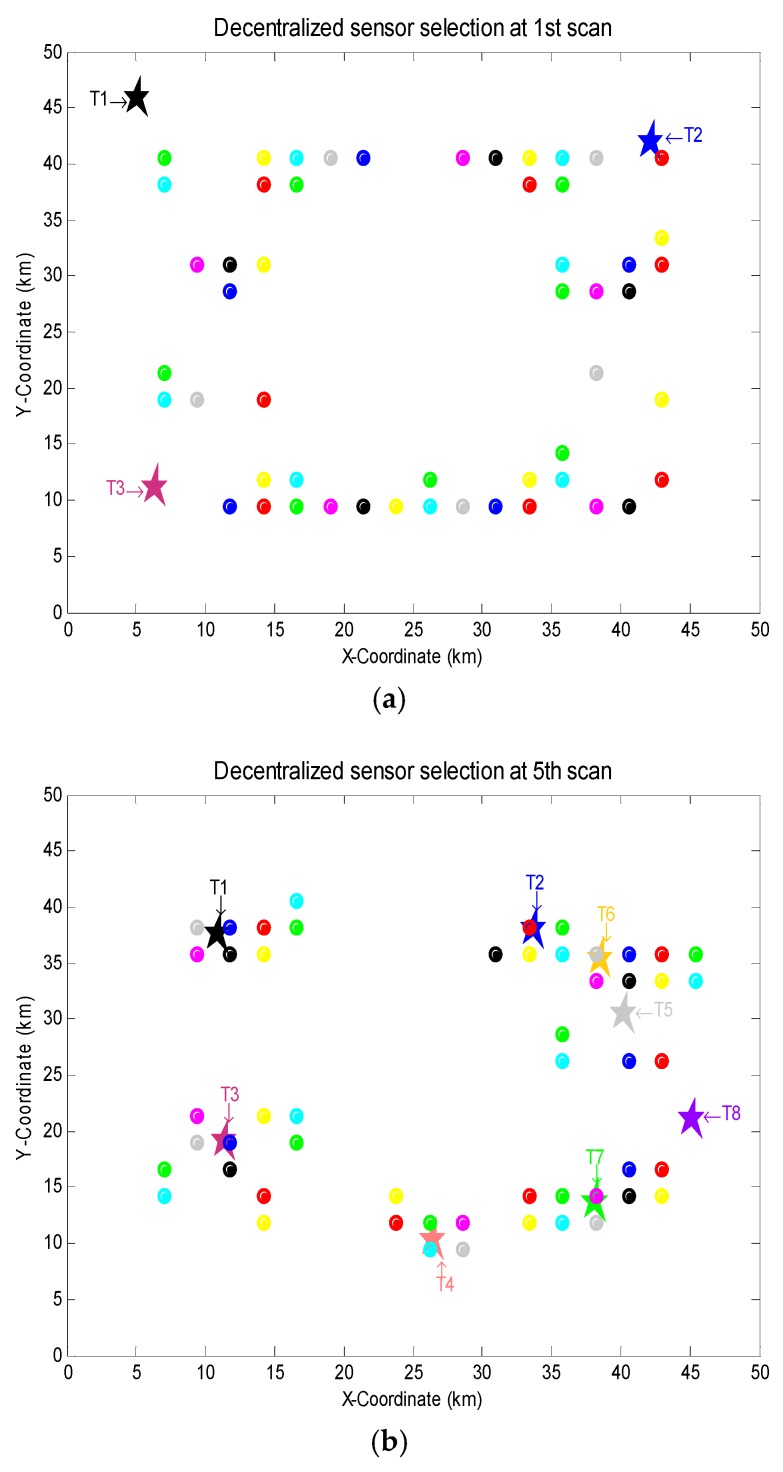

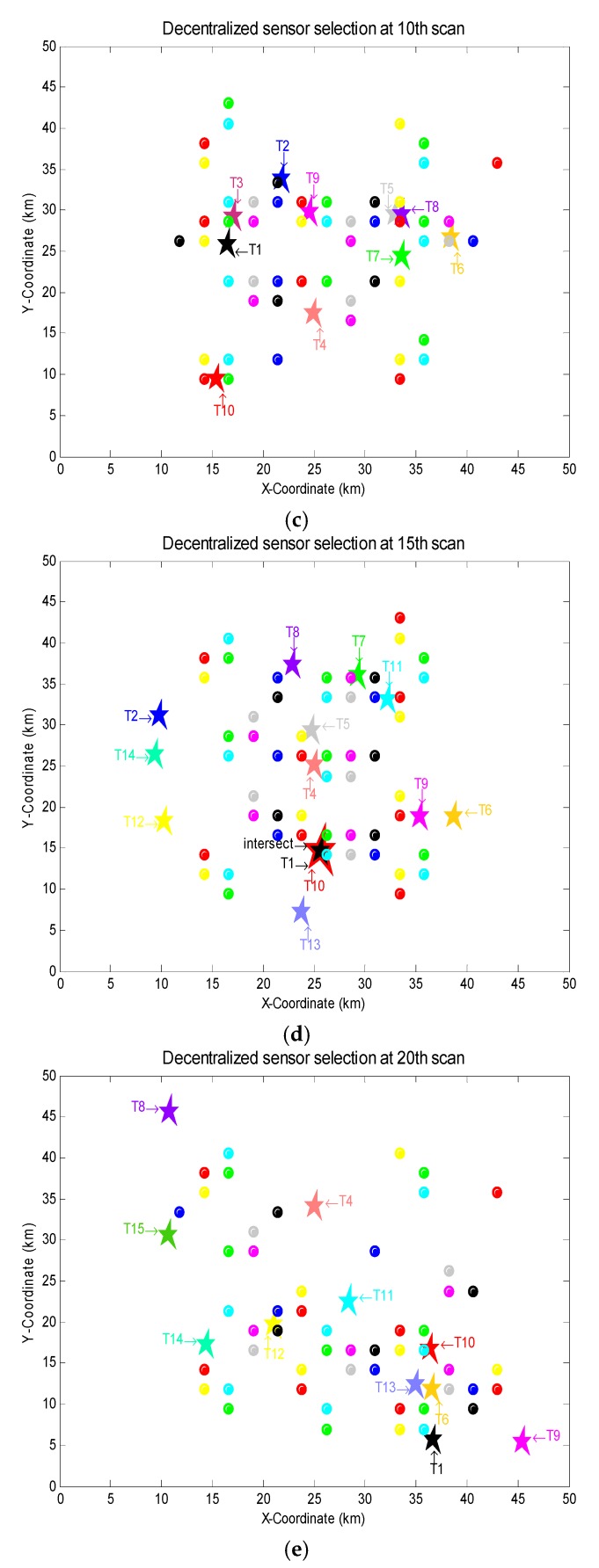

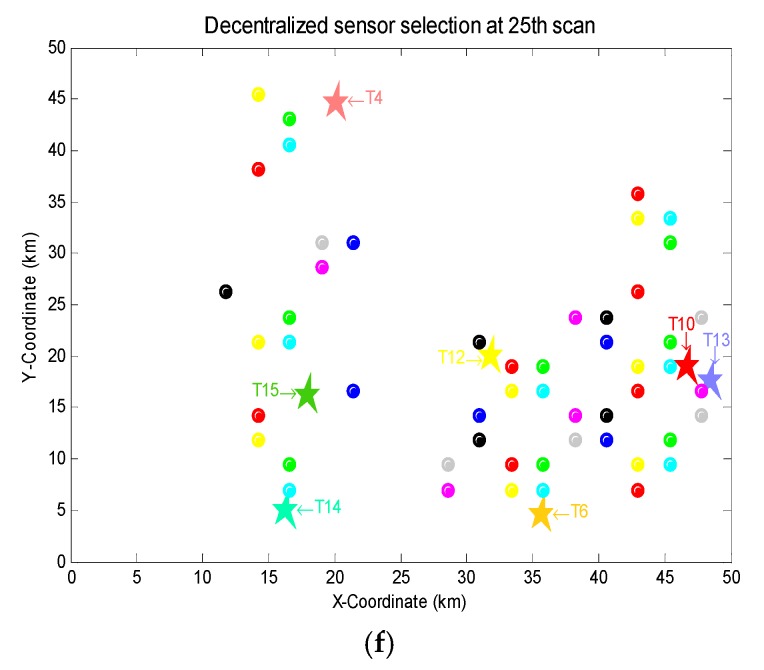
Sensor selection using ***LA bound with coordinate descend*** at (**a**) the 1st scan; (**b**) the 5th scan; (**c**) the 10th scan; (**d**) the 15th scan; (**e**) the 20th scan; (**f**) the 25th scan. ★ and ☉ denote the targets and selected SNs, different colors correspond to different targets and SNs of different LFCs.

Figure 4a~f show the results of sensor selection at the 1st, 5th, 10th, 15th, 20th and 25th scans. It can be seen that the SNs selected by each LFC will change adaptively with the multi-target movement versus time. Specifically, in order to minimize the bound in Theorem 1, most of the selected SNs locate in the regions closer to the survival targets at each scan. A few SNs which are far from the survival targets are selected to satisfy Constraint **C2**. Moreover, due to Constraint **C3**, the homogeneous SNs of each LFC cannot be excessively concentrated in a small region.

In order to further verify the performance of this method in tracking accuracy and computational time, it is compared with the methods of ***LA bound with genetic algorithm***, ***CS divergence with genetic algorithm*** and ***Random selection*** under the same test platform. In the genetic algorithm, the population size is 50, crossing rate is 0.9, mutation rate is 0.001, elite rate is 0.04 and the maximum number of iterations is 500. In the CS divergence method, the objective function of the LFC j is

(82) $${\left[S^{j}\right]}^{*}=\underset{S^{j}\subseteq N^{j}}{\arg\max}\;E\left[D_{CS}\left(\pi_{+}^{j},\pi^{j}\left(\cdot |Z^{S^{j}}\right)\right)\right]\;$$

where $D_{CS}\left(\phi ,\varphi \right)$ denotes the CS divergence between the densities ϕ and φ. [31] presents the specific form of the CS divergence when ϕ and φ are both GLMB densities. Since the posterior density $\pi^{j}\left(X|Z^{S^{j}}\right)$ is unknown before sensor selection, the expected value rather than real value of the CS divergence is applied in (82). In order to calculate the expected value, the MC integration based on PIMS also needs to be used here.

For comparison, both the OSPA and LA metrics are used to measure the error of multi-target position estimates.

**Figure 5:**
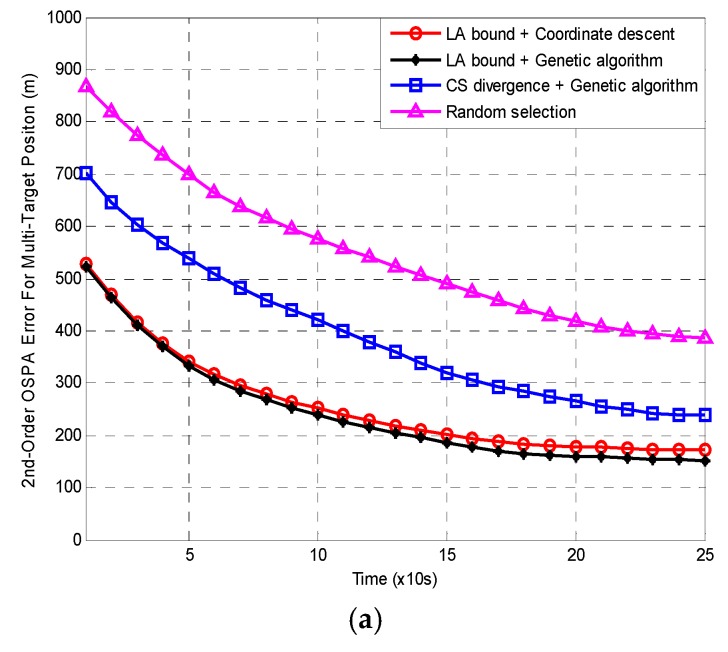

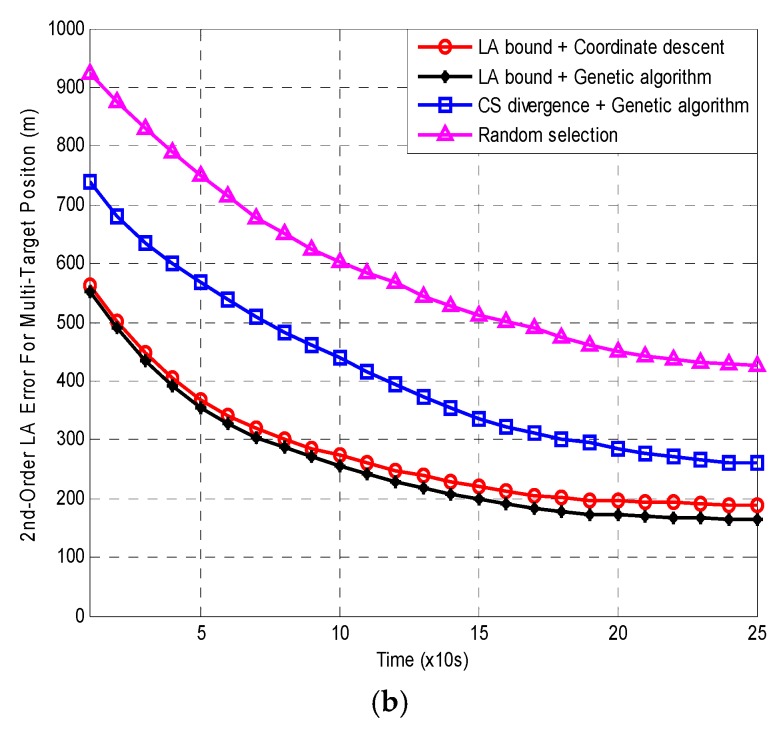
500 MC averages of (**a**) OSPA and (**b**) LA errors versus time with c=1000 m.

Figure 5a,b present the 500 MC averages of the OSPA and LA errors for the four methods. Note that the error here is selected as the average of all LFCs because of Remark 1.

Figure 5 shows that both the averaged OSPA and LA errors from all the four methods decrease with time. Furthermore, the LA errors are always larger than the relevant OSPA errors. The reason for this has been explained in Section 2.3. In both of Figure 5a,b, the errors from ***Random selection*** are always the largest. The next is the errors from ***CS divergence with genetic algorithm***. The errors from ***LA bound with genetic algorithm*** are always the smallest. The errors from ***LA bound with coordinate descent*** are slightly larger than those of ***LA bound with genetic algorithm***. Compared with ***Random selection***, the errors of the other three algorithms are approximately reduced by 40%, 60% and 55%, respectively. Obviously, the two LA bound based methods outperform the CS divergence based method in MTT accuracy. There are three reasons for this:

1) The LA bound has a clearer physical meaning than the CS divergence. This is because that the former indicates the achievable optimal MTT accuracy with labeled RFS state. In contrast, the latter is not directly related to the MTT accuracy since maximizing the CS divergence in (82) cannot guarantee to minimize the OSPA or LA error.

2) The CS divergence cannot provide a basis for setting the weights of KLA rule as the LA bound. Therefore, in the CS divergence based method, the KLA weights can only be set to the same by convention [32]. However, in this example the MTT accuracy of different LFCs is probably not the same because of the distinguishing observation performance of the diverse SNs. If the KLA weights are set to the same without discrimination, the fusion efficiency will decline dramatically in this case.

3) In the step of sensor selection optimization, the coordinate descent method may trap in one of local optimums. In contrast, the genetic algorithm may jump out of local optimums with certain probability by its randomness. This leads that the MTT accuracy of the former is a litter worse than the latter.

On the other hand, the computational cost of a method is in general measured by its CPU run time. Then, the averaged CPU run time per scan for ***LA bound with coordinate descent***, ***LA bound with genetic algorithm*** and ***CS divergence with genetic algorithm*** are 0.62s, 6.18s and 5.69s, respectively. Note that the run time here is also selected as the average of all LFCs because of Remark 1. ***Random selection*** obviously does not need the optimization for sensor selection, so it has no time consumption of this step. It can be seen from this that although the coordinate descent method is slightly worse than the genetic algorithm in tracking accuracy, it significantly shortens the calculation time of sensor selection optimization. In addition, the time consumption of ***CS divergence with genetic algorithm*** is slightly less than that of ***LA bound with genetic algorithm***. This case indicates that the computational cost of the proposed bound is a little larger than that of the CS divergence expectation in (82).

In order to show the influence of different SNR on our method, the clutter rate and detection probability of each SN are changed into $\left(\lambda =40,p_{d}=0.85\right)$, $\left(\lambda =60,p_{d}=0.75\right)$, $\left(\lambda =80,p_{d}=0.65\right)$ and $\left(\lambda =100,p_{d}=0.55\right)$. Table 1 and Table 2 present the final values of the OSPA and LA errors for the four methods in each scenario after 500 MC run average.

Moreover, the CPU times consumed by the sensor selection optimization for the first three methods are nearly the same for all the SNR scenarios. This is because that the sensor selection is irrelevant to the specific measurement realizations since it must be completed before the measurements are received.

It can be seen from Algorithm 1 and 2 that as clutter density increases and detection probability decreases,

1) The OSPA and LA errors of all the four methods increase in different degrees but the size order of them is always the same as that of Scenario 1;

2) Taking the error of ***Random selection*** as the benchmark, the improvement ratio of ***CS divergence with genetic algorithm*** is gradually reduced from about 40% to about 20%. Meanwhile, the improvement ratios of the two LA bound based methods are, respectively, maintained at about 60% and 55%.

The two points reflect that the lower the SNR is, the worse the sensor selection efficiency and tracking accuracy of the CS divergence based methods become. By contrast, the tracking accuracy of the LA bound based methods always maintains a good improvement ratio for all the SNR scenarios.

Assuming that the simulation scenarios remain unchanged, the above four methods are re-implemented by the GM technique where the non-linear likelihood of each SN is approximated by the EK filter,

(83) $${\hat{H}}^{s}{\approx \frac{\partial h^{s}(x)}{\partial x}|}_{x={\hat{x}}_{+}};\;{\hat{R}}^{s}\approx R^{s}\left({\hat{x}}_{+}\right)\;$$

The pruning and merging technology [11] are used to manage the GM terms. Let the number of the GM terms approximating to each $p_{I}\left(\cdot ,\ell \right)$ be no more than 30. The thresholds for merging, pruning and state extraction are set to 4, $10^{-4}$ and 0.5, respectively. The simulation results of the GM implementation are basically consistent with those of the SMC implementation, except that the OSPA or LA error increases by about 8%. But the calculation time for sensor selection decreases by about 70%.

## 6. Conclusions and Future Work

A sensor selection optimization algorithm is proposed for the decentralized large-scale MTT network under the labeled RFS framework. The LA metric defined in this paper is used to measure the error between the labeled multi-target states and their estimates. The lower bound of the LA metric based MSE is taken as the cost function of sensor selection. The bound is derived by the information inequality and then, implemented by the SMC or GM technique. Then, the coordinate descent method is used to reduce the computational cost of sensor selection. Simulation results show that when the sensors of the decentralized network have different observation performance, our method outperforms the CS divergence based sensor selection algorithm in MTT accuracy.

Our future work will focus on the following two aspects:

1) Extend the proposed method to the cases of asynchronous measurement or correlated measurement noise;

2) Reference [50] has presented a very efficient implementation of the GLMB filter with linear complexity in the number of measurements and this filter has been demonstrated to handle over 1 million tracks simultaneously [51]. Therefore, it would be very helpful to improve our current study by the use of the methods proposed in Reference [50,51].

## Figures and Tables

**Table 1:** Final value of OSPA error (Unit: m).

	Clutter rate and Detection Probability	$\begin{array}{c} \lambda =20\\ p_{d}=0.95 \end{array}\;$	$\begin{array}{c} \lambda =40\\ p_{d}=0.85 \end{array}\;$	$\begin{array}{c} \lambda =60\\ p_{d}=0.75 \end{array}\;$	$\begin{array}{c} \lambda =80\\ p_{d}=0.65 \end{array}\;$	$\begin{array}{c} \lambda =100\\ p_{d}=0.55 \end{array}\;$
Sensor Selection Method						
***LA bound with coordinate descent***		171.3	193.0	218.9	249.1	283.7
***LA bound with genetic algorithm***		151.9	170.8	193.6	220.5	251.6
***CS divergence with genetic algorithm***		238.6	278.5	333.7	405.3	494.4
***Random selection***		386.7	436.0	490.5	550.1	615.2

**Table 2:** Final value of LA error (Unit: m).

	Clutter rate and Detection Probability	$\begin{array}{c} \lambda =20\\ p_{d}=0.95 \end{array}\;$	$\begin{array}{c} \lambda =40\\ p_{d}=0.85 \end{array}\;$	$\begin{array}{c} \lambda =60\\ p_{d}=0.75 \end{array}\;$	$\begin{array}{c} \lambda =80\\ p_{d}=0.65 \end{array}\;$	$\begin{array}{c} \lambda =100\\ p_{d}=0.55 \end{array}\;$
Sensor Selection Method						
***LA bound with coordinate descent***		188.8	223.9	255.0	288.6	323.9
***LA bound with genetic algorithm***		165.7	199.6	229.5	262.9	292.3
***CS divergence with genetic algorithm***		260.1	321.2	383.9	463.9	562.8
***Random selection***		426.7	500.5	564.3	634.2	710.9

## Appendix A.

**Proof that** ${\bar{d}}_{p}^{\left(c\right)}\left(X,Y\right)$ **is a metric**

A mapping d:X×X→[0,∞) is called a metric if it obeys the following three properties

**1) Identity:**d(x,y)=0**if and only if**x=y;

**2) Symmetry:**d(x,y)=d(y,x)**for all**x,y∈X;

**3) Triangle inequality:**d(x,y)≤d(x,z)+d(z,y)**for all**x,y,z∈X.

It can be obtained easily from the definition of ${\bar{d}}_{p}^{\left(c\right)}\left(X,Y\right)$ in (9) that $0\leq {\bar{d}}_{p}^{\left(c\right)}\left(X,Y\right)\leq c$ and ${\bar{d}}_{p}^{\left(c\right)}\left(X,Y\right)$ obeys the properties of identity and symmetry. Next, we will prove the triangle inequality

(A1) $${\bar{d}}_{p}^{\left(c\right)}\left(X,Y\right)\leq {\bar{d}}_{p}^{\left(c\right)}\left(X,Z\right)+{\bar{d}}_{p}^{\left(c\right)}\left(Z,Y\right)\;$$

Let x,y,z and ℒ(X),ℒ(Y),ℒ(Z) denote the individual elements and label sets of X,Y,Z, respectively. We firstly set

(A2) $$\begin{array}{cc} x_{\ell }:=u_{\ell } & \mathrm{for}\;\ell \in ℒ(X)\cup ℒ(Y)\cup ℒ(Z)-ℒ(X) \end{array}\;$$

(A3) $$\begin{array}{cc} y_{\ell }:=v_{\ell } & \mathrm{for}\;\ell \in ℒ(X)\cup ℒ(Y)\cup ℒ(Z)-ℒ(Y) \end{array}\;$$

(A4) $$\left\{ \begin{array}{l} \begin{array}{cc} z_{\ell }:=u_{\ell } & \mathrm{for}\;\ell \in ℒ(X)\cup ℒ(Y)\cup ℒ(Z)-ℒ(X)\cup ℒ(Z) \end{array}\\ \begin{array}{cc} z_{\ell }:=v_{\ell } & \mathrm{for}\;\ell \in ℒ(X)\cup ℒ(Z)-ℒ(Z) \end{array} \end{array}\right.$$

where $u_{\ell }$ and $v_{\ell }$ satisfy

(A5) $$\left\|u_{\ell }-w\right\|\geq c,\;\left\|v_{\ell }-w\right\|\geq c\;\mathrm{and}\;\left\|u_{\ell }-v_{\ell^{\prime }}\right\|\geq c\;$$

for all w∈X∪Y∪Z and all $\ell ,\ell^{\prime }\in ℒ(X)\cup ℒ(Y)\cup ℒ(Z)$.

Based on the setting of (A2)–(A5), the definition of ${\bar{d}}_{p}^{\left(c\right)}\left(X,Y\right)$ in (9) is rewritten as

(A6) $${\bar{d}}_{p}^{\left(c\right)}\left(X,Y\right)={\left(\frac{\sum_{\ell \in ℒ(X)\cup ℒ(Y)}d^{\left(c\right)}{\left(x_{\ell },y_{\ell }\right)}^{p}}{\left|ℒ(X)\cup ℒ(Y)\right|}\right)}^{\frac{1}{p}}\;$$

We will prove that the triangle inequality of (A1) holds for all six possible ranks of |ℒ(X)∪ℒ(Y)|, |ℒ(X)∪ℒ(Z)| and |ℒ(Z)∪ℒ(Y)|.

**Case 1** (|ℒ(X)∪ℒ(Y)|≤|ℒ(X)∪ℒ(Z)|≤|ℒ(Y)∪ℒ(Z)|): Since the cut-off distance $d^{\left(c\right)}\left(x_{\ell },y_{\ell }\right)\leq c$ for all ℓ∈ℒ(X)∪ℒ(Y)∪ℒ(Z) and |ℒ(X)∪ℒ(Y)|≤|ℒ(X)∪ℒ(Z)|, (A6) can be amplified to

(A7) $$\begin{array}{ll} {\bar{d}}_{p}^{\left(c\right)}\left(X,Y\right) & \leq {\left(\frac{\sum_{\ell \in ℒ(X)\cup ℒ(Z)}d^{\left(c\right)}{\left(x_{\ell },y_{\ell }\right)}^{p}}{\left|ℒ(X)\cup ℒ(Z)\right|}\right)}^{\frac{1}{p}}\\ & \leq {\left(\frac{\sum_{\ell \in ℒ(X)\cup ℒ(Z)}{\left(d^{\left(c\right)}\left(x_{\ell },z_{\ell }\right)+d^{\left(c\right)}\left(z_{\ell },y_{\ell }\right)\right)}^{p}}{\left|ℒ(X)\cup ℒ(Z)\right|}\right)}^{\frac{1}{p}} \end{array}$$

where the last line holds because $d^{\left(c\right)}\left(x_{\ell },y_{\ell }\right)$ obeys the triangle inequality.

Applying Minkowski’s inequality to (A7), we get

(A8) $$\begin{array}{ll} {\bar{d}}_{p}^{\left(c\right)}\left(X,Y\right) & \leq {\left(\frac{\sum_{\ell \in ℒ(X)\cup ℒ(Z)}d^{\left(c\right)}{\left(x_{\ell },z_{\ell }\right)}^{p}}{\left|ℒ(X)\cup ℒ(Z)\right|}\right)}^{\frac{1}{p}}+{\left(\frac{\sum_{\ell \in ℒ(X)\cup ℒ(Z)}d^{\left(c\right)}{\left(z_{\ell },y_{\ell }\right)}^{p}}{\left|ℒ(X)\cup ℒ(Z)\right|}\right)}^{\frac{1}{p}}\\ & ={\bar{d}}_{p}^{\left(c\right)}\left(X,Z\right)+{\left(\frac{\sum_{\ell \in ℒ(X)\cup ℒ(Z)}d^{\left(c\right)}{\left(z_{\ell },y_{\ell }\right)}^{p}}{\left|ℒ(X)\cup ℒ(Z)\right|}\right)}^{\frac{1}{p}} \end{array}$$

Similar with the derivation of the first line in (A7), the second term on the right-hand side of ‘=’ in (A8) can be amplified to

(A9) $$\begin{array}{ll} {\left(\frac{\sum_{\ell \in ℒ(X)\cup ℒ(Z)}d^{\left(c\right)}{\left(z_{\ell },y_{\ell }\right)}^{p}}{\left|ℒ(X)\cup ℒ(Z)\right|}\right)}^{\frac{1}{p}} & \leq {\left(\frac{\sum_{\ell \in ℒ(Y)\cup ℒ(Z)}d^{\left(c\right)}{\left(z_{\ell },y_{\ell }\right)}^{p}}{\left|ℒ(Y)\cup ℒ(Z)\right|}\right)}^{\frac{1}{p}}\\ & ={\bar{d}}_{p}^{\left(c\right)}\left(Y,Z\right) \end{array}$$

because of $d^{\left(c\right)}\left(x_{\ell },y_{\ell }\right)\leq c$ and |ℒ(X)∪ℒ(Z)|≤|ℒ(Y)∪ℒ(Z)|.

Finally, the triangle inequality of (A1) for Case 1 is obtained by substituting (A9) into (A8).

**Case 2 (** |ℒ(X)∪ℒ(Y)|≤|ℒ(Y)∪ℒ(Z)|≤|ℒ(X)∪ℒ(Z)| **):**

The proof of Case 2 is similar with that of Case 1 except that its first amplification is completed by using |ℒ(X)∪ℒ(Y)|≤|ℒ(Y)∪ℒ(Z)| and its last amplification is completed by using |ℒ(Y)∪ℒ(Z)|≤|ℒ(X)∪ℒ(Z)|.

**Case 3 (** |ℒ(X)∪ℒ(Z)|≤|ℒ(Y)∪ℒ(Z)|≤|ℒ(X)∪ℒ(Y)| **):**

Using the triangle inequality of $d^{\left(c\right)}\left(x_{\ell },y_{\ell }\right)$, (A6) can be amplified to

(A10) $$\begin{array}{ll} {\bar{d}}_{p}^{\left(c\right)}\left(X,Y\right) & \leq {\left(\frac{\sum_{\ell \in ℒ(X)\cup ℒ(Y)}{\left(d^{\left(c\right)}\left(x_{\ell },z_{\ell }\right)+d^{\left(c\right)}\left(z_{\ell },y_{\ell }\right)\right)}^{p}}{\left|ℒ(X)\cup ℒ(Y)\right|}\right)}^{\frac{1}{p}}\\ & \leq {\left(\frac{\sum_{\ell \in ℒ(X)\cup ℒ(Y)}{\left(d^{\left(c\right)}\left(x_{\ell },z_{\ell }\right)\right)}^{p}}{\left|ℒ(X)\cup ℒ(Y)\right|}\right)}^{\frac{1}{p}}+{\left(\frac{\sum_{\ell \in ℒ(X)\cup ℒ(Y)}{\left(d^{\left(c\right)}\left(z_{\ell },y_{\ell }\right)\right)}^{p}}{\left|ℒ(X)\cup ℒ(Y)\right|}\right)}^{\frac{1}{p}} \end{array}$$

where the last line is derived from Minkowski’s inequality.

Because of |ℒ(X)∪ℒ(Z)|≤|ℒ(Y)∪ℒ(Z)|≤|ℒ(X)∪ℒ(Y)|, (A10) can be amplified to

(A11) $${\bar{d}}_{p}^{\left(c\right)}\left(X,Y\right)\leq {\left(\frac{\sum_{\ell \in ℒ(X)\cup ℒ(Y)}{\left(d^{\left(c\right)}\left(x_{\ell },z_{\ell }\right)\right)}^{p}}{\left|ℒ(X)\cup ℒ(Z)\right|}\right)}^{\frac{1}{p}}+{\left(\frac{\sum_{\ell \in ℒ(X)\cup ℒ(Y)}{\left(d^{\left(c\right)}\left(z_{\ell },y_{\ell }\right)\right)}^{p}}{\left|ℒ(Z)\cup ℒ(Y)\right|}\right)}^{\frac{1}{p}}\;$$

Then, based on the setting of (A2)–(A5), we have

(A12) $$\left\{ \begin{array}{l} \sum_{\ell \in ℒ(X)\cup ℒ(Y)}{\left(d^{\left(c\right)}\left(x_{\ell },z_{\ell }\right)\right)}^{p}\leq \sum_{\ell \in ℒ(X)\cup ℒ(Z)}{\left(d^{\left(c\right)}\left(x_{\ell },z_{\ell }\right)\right)}^{p}\\ \sum_{\ell \in ℒ(X)\cup ℒ(Y)}{\left(d^{\left(c\right)}\left(z_{\ell },y_{\ell }\right)\right)}^{p}\leq \sum_{\ell \in ℒ(Z)\cup ℒ(Y)}{\left(d^{\left(c\right)}\left(z_{\ell },y_{\ell }\right)\right)}^{p} \end{array}\right.\;$$

Substituting (A12) into (A11), (A11) can be amplified to

(A13) $$\begin{array}{ll} {\bar{d}}_{p}^{\left(c\right)}\left(X,Y\right) & \leq {\left(\frac{\sum_{\ell \in ℒ(X)\cup ℒ(Z)}{\left(d^{\left(c\right)}\left(x_{\ell },z_{\ell }\right)\right)}^{p}}{\left|ℒ(X)\cup ℒ(Z)\right|}\right)}^{\frac{1}{p}}+{\left(\frac{\sum_{\ell \in ℒ(Z)\cup ℒ(Y)}{\left(d^{\left(c\right)}\left(z_{\ell },y_{\ell }\right)\right)}^{p}}{\left|ℒ(Z)\cup ℒ(Y)\right|}\right)}^{\frac{1}{p}}\\ & ={\bar{d}}_{p}^{\left(c\right)}\left(X,Z\right)+{\bar{d}}_{p}^{\left(c\right)}\left(Z,Y\right) \end{array}$$

which is just the triangle inequality of (A1) for Case 3.

**Case 4 (** |ℒ(Y)∪ℒ(Z)|≤|ℒ(X)∪ℒ(Z)|≤|ℒ(X)∪ℒ(Y)| **):**

The proof of Case 4 is exactly the same as that of Case 3.

**Case 5 (** |ℒ(Y)∪ℒ(Z)|≤|ℒ(X)∪ℒ(Y)|≤|ℒ(X)∪ℒ(Z)| **):**

First, (A10) still holds for Case 5 depending on the triangle inequality of $d^{\left(c\right)}\left(x_{\ell },y_{\ell }\right)$ and Minkowski’s inequality.

Since $d^{\left(c\right)}\left(x_{\ell },y_{\ell }\right)\leq c$ for all ℓ∈ℒ(X)∪ℒ(Y)∪ℒ(Z) and |ℒ(X)∪ℒ(Y)|≤|ℒ(X)∪ℒ(Z)|, we have

(A14) $$\begin{array}{ll} {\left(\frac{\sum_{\ell \in ℒ(X)\cup ℒ(Y)}{\left(d^{\left(c\right)}\left(x_{\ell },z_{\ell }\right)\right)}^{p}}{\left|ℒ(X)\cup ℒ(Y)\right|}\right)}^{\frac{1}{p}} & \leq {\left(\frac{\sum_{\ell \in ℒ(X)\cup ℒ(Z)}{\left(d^{\left(c\right)}\left(x_{\ell },z_{\ell }\right)\right)}^{p}}{\left|ℒ(X)\cup ℒ(Z)\right|}\right)}^{\frac{1}{p}}\\ & ={\bar{d}}_{p}^{\left(c\right)}\left(X,Z\right) \end{array}$$

From |ℒ(Y)∪ℒ(Z)|≤|ℒ(X)∪ℒ(Y)|, we have

(A15) $$\begin{array}{ll} {\left(\frac{\sum_{\ell \in ℒ(X)\cup ℒ(Y)}{\left(d^{\left(c\right)}\left(z_{\ell },y_{\ell }\right)\right)}^{p}}{\left|ℒ(X)\cup ℒ(Y)\right|}\right)}^{\frac{1}{p}} & \leq {\left(\frac{\sum_{\ell \in ℒ(X)\cup ℒ(Y)}{\left(d^{\left(c\right)}\left(z_{\ell },y_{\ell }\right)\right)}^{p}}{\left|ℒ(Z)\cup ℒ(Y)\right|}\right)}^{\frac{1}{p}}\\ & \leq {\left(\frac{\sum_{\ell \in ℒ(Z)\cup ℒ(Y)}{\left(d^{\left(c\right)}\left(z_{\ell },y_{\ell }\right)\right)}^{p}}{\left|ℒ(Z)\cup ℒ(Y)\right|}\right)}^{\frac{1}{p}}\\ & ={\bar{d}}_{p}^{\left(c\right)}\left(Z,Y\right) \end{array}$$

where the second line is obtained due to (A12).

Substituting (A14) and (A15) into (A10), we finally obtain the triangle inequality of (A1) for Case 5.

**Case 6 (** |ℒ(X)∪ℒ(Z)|≤|ℒ(X)∪ℒ(Y)|≤|ℒ(Y)∪ℒ(Z)| **):**

The proof of Case 6 is similar with that of Case 5, except that (A14) is derived from |ℒ(X)∪ℒ(Z)|≤|ℒ(X)∪ℒ(Y)| and (A12) while (A15) is derived from $d^{\left(c\right)}\left(x_{\ell },y_{\ell }\right)\leq c$ and |ℒ(X)∪ℒ(Y)|≤|ℒ(Y)∪ℒ(Z)|.

Above all, the proof that ${\bar{d}}_{p}^{\left(c\right)}\left(X,Y\right)$ in (9) is a metric has been completed. □

## Appendix B.

**Proof of Theorem 1**

From (4) and (18), $\sigma_{S}^{2}$ in (16) is written as

(A16) $$\sigma_{S}^{2}=\sum_{m^{S}=0}^{\infty }\sum_{n=0}^{\infty }\frac{\Omega_{n,m^{S}}}{m^{S}!n!}\sum_{\ell_{1:n}\in L_{n}}\int_{{\mathbb{Z}}_{m^{S}}^{S}}\int_{X_{n}}q\left(X_{n},Z_{m^{S}}^{S}\right)e^{2}\left(X_{n},\hat{X}\left(Z_{m^{S}}^{S}\right)\right)dx_{1:n}dz_{1:m^{S}}^{S}\;$$

where $q\left(X_{n},Z_{m^{S}}^{S}\right)$ and $\Omega_{n,m^{S}}$ are defined in (18) and (19), $\Omega_{n,m^{S}}$ is finally obtained as (24).

Dividing the integral region ${\mathbb{Z}}_{m^{S}}^{S}$ of (A16) into ${\mathbb{Z}}_{0,m^{S}}^{S},{\mathbb{Z}}_{1,m^{S}}^{S},\ldots ,{\mathbb{Z}}_{\infty ,m^{S}}^{S}$ according to (30), $\sigma_{S}^{2}$ is rewritten as

(A17) $$\sigma_{S}^{2}=\sum_{m^{S}=0}^{\infty }\sum_{n=0}^{\infty }\frac{\Omega_{n,m^{S}}}{m^{S}!n!}\sum_{\hat{n}=0}^{\infty }\sum_{\ell_{1:n}\in L_{n}}\int_{{\mathbb{Z}}_{\hat{n},m^{S}}^{S}}\int_{X_{n}}q\left(X_{n},Z_{m^{S}}^{S}\right)e^{2}\left(X_{n},{\hat{X}}_{\hat{n}}\left(Z_{m^{S}}^{S}\right)\right)dx_{1:n}dz_{1:m^{S}}^{S}\;$$

where the error $e\left(X_{n},{\hat{X}}_{\hat{n}}\left(Z_{m^{S}}^{S}\right)\right)$ is measured by the 2nd-order LA metric ${\bar{d}}_{2}^{\left(c\right)}\left(X_{n},{\hat{X}}_{\hat{n}}\left(Z_{m^{S}}^{S}\right)\right)$ defined in (9). Substituting ${\bar{d}}_{2}^{\left(c\right)}\left(X_{n},{\hat{X}}_{\hat{n}}\left(Z_{m^{S}}^{S}\right)\right)$ into (A17) and then using the identical equation |ℒ(X)∪ℒ(Y)|=|ℒ(X)|+|ℒ(Y)|−|ℒ(X)∩ℒ(Y)|, we get

(A18) $$\begin{array}{ll} \sigma_{S}^{2} & =\sum_{m^{S}=0}^{\infty }\sum_{n=0}^{\infty }\frac{\Omega_{n,m^{S}}}{m^{S}!n!}\sum_{\hat{n}=0,n+\hat{n}>0}^{\infty }\sum_{\ell_{1:n}\in L_{n}}\frac{1}{n+\hat{n}-\left|ℒ\left(X_{n}\right)\cap ℒ\left({\hat{X}}_{\hat{n}}\left(Z_{m^{S}}^{S}\right)\right)\right|}\int_{{\mathbb{Z}}_{\hat{n},m^{S}}^{S}}\int_{X_{n}}q\left(X_{n},Z_{m^{S}}^{S}\right)\\ & \cdot \left(\sum_{\ell^{\prime }\in ℒ(X_{n})\cap ℒ\left({\hat{X}}_{\hat{n}}\left(Z_{m^{S}}^{S}\right)\right)}\min\left(c^{2},{\left\|x_{\ell^{\prime }}-{\hat{x}}_{\ell^{\prime }}\left(Z_{m^{S}}^{S}\right)\right\|}^{2}\right)+c^{2}\left(n+\hat{n}-2\left|ℒ\left(X_{n}\right)\cap ℒ\left({\hat{X}}_{\hat{n}}\left(Z_{m^{S}}^{S}\right)\right)\right|\right)\right)dx_{1:n}dz_{1:m^{S}}^{S} \end{array}$$

where $\ell_{1:n}$ are the labels of $X_{n}$. It is obvious that $ℒ\left(X_{n}\right)\cap ℒ\left({\hat{X}}_{\hat{n}}\left(Z_{m^{S}}^{S}\right)\right)\subseteq \left\{\ell_{1:n}\right\}$.

Let $k=\left|ℒ\left(X_{n}\right)\cap ℒ\left({\hat{X}}_{\hat{n}}\left(Z_{m^{S}}^{S}\right)\right)\right|$. $0\leq k\leq \min\left(n,\hat{n}\right)$. Given k and $\ell_{1:n}$, there are $C_{n}^{k}=n!/\left(k!\left(n-k\right)!\right)$ possible choices from $\ell_{1:n}$ for the elements of $\left\{{\ell^{\prime }}_{1:k}\right\}=ℒ\left(X_{n}\right)\cap ℒ\left({\hat{X}}_{\hat{n}}\left(Z_{m^{S}}^{S}\right)\right)$. Then, (A18) can be rewritten as

(A19) $$\begin{array}{ll} \sigma_{S}^{2} & =\sum_{m^{S}=0}^{\infty }\sum_{n=0}^{\infty }\sum_{\hat{n}=0,n+\hat{n}>0}^{\infty }\sum_{\ell_{1:n}\in L_{n}}\sum_{k=0}^{\min(n,\hat{n})}\sum_{\left\{{\ell^{\prime }}_{1:k}\}\in \{\ell_{1:n}\right\}}\frac{\Omega_{n,m^{S}}}{m^{S}!n!}\cdot \frac{1}{n+\hat{n}-k}\int_{{\mathbb{Z}}_{\hat{n},m^{S}}^{S}}\int_{X_{n}}q\left(X_{n},Z_{m^{S}}^{S}\right)\\ & \cdot \left(\sum_{\ell^{\prime }\in \{{\ell^{\prime }}_{1:k}\}}\min\left(c^{2},{\left\|x_{\ell^{\prime }}-{\hat{x}}_{\ell^{\prime }}\left(Z_{m^{S}}^{S}\right)\right\|}^{2}\right)+c^{2}\left(n+\hat{n}-2k\right)\right)dx_{1:n}dz_{1:m^{S}}^{S}\\ & =\sum_{m^{S}=0}^{\infty }\sum_{n=0}^{\infty }\sum_{\hat{n}=0,n+\hat{n}>0}^{\infty }\sum_{k=0}^{\min(n,\hat{n})}\sum_{{\ell^{\prime }}_{1:k}\in L_{k}}\frac{\Omega_{n,m^{S}}\Psi_{\hat{n},n,m^{S}}}{m^{S}!k!\left(n-k\right)!}\cdot \frac{1}{n+\hat{n}-k}\\ & \cdot \left(\sum_{\ell^{\prime }\in \{{\ell^{\prime }}_{1:k}\}}\min\left(c^{2},\frac{1}{\Psi_{\hat{n},n,m^{S}}}\sum_{\left(\ell_{1:n}-\ell^{\prime }\right)\in L_{n-1}}\int_{{\mathbb{Z}}_{\hat{n},m^{S}}^{S}}\int_{X_{n}}q\left(X_{n},Z_{m^{S}}^{S}\right){\left\|x_{\ell^{\prime }}-{\hat{x}}_{\ell^{\prime }}\left(Z_{m^{S}}^{S}\right)\right\|}^{2}dx_{1:n}dz_{1:m^{S}}^{S}\right)+c^{2}\left(n+\hat{n}-2k\right)\right) \end{array}$$

where $\Psi_{\hat{n},n,m^{S}}$ is defined in (34) and finally obtained as (39), $\ell_{1:n}-\ell^{\prime }$ denotes the residual label sequence of $\ell_{1:n}$ after the label $\ell^{\prime }$ is separately excluded from $\ell_{1:n}$.

Note that the estimate ${\hat{x}}_{\ell^{\prime }}\left(Z_{m^{S}}^{S}\right)$ involved in (A19) is independent of $X_{n}$. So, the integral term in the last line of (A19) is obtained as

(A20) $$\begin{array}{l} \sum_{\left(\ell_{1:n}-\ell^{\prime }\right)\in L_{n-1}}\int_{{\mathbb{Z}}_{\hat{n},m^{S}}^{S}}\int_{X_{n}}q\left(X_{n},Z_{m^{S}}^{S}\right){\left\|x_{\ell^{\prime }}-{\hat{x}}_{\ell^{\prime }}\left(Z_{m^{S}}^{S}\right)\right\|}^{2}dx_{1:n}dz_{1:m^{S}}^{S}\\ =\int_{{\mathbb{Z}}_{\hat{n},m^{S}}^{S}}\int_{X_{1}}\left[\sum_{\left(\ell_{1:n}-\ell^{\prime }\right)\in L_{n-1}}\int_{X_{n-1}}q\left(X_{n},Z_{m^{S}}^{S}\right)d\left(x_{1:n}-x_{\ell^{\prime }}\right)\right]{\left\|x_{\ell^{\prime }}-{\hat{x}}_{\ell^{\prime }}\left(Z_{m^{S}}^{S}\right)\right\|}^{2}dx_{\ell^{\prime }}dz_{1:m^{S}}^{S}\\ =\int_{{\mathbb{Z}}_{\hat{n},m^{S}}^{S}}\int_{X_{1}}q_{n}\left(x,\ell^{\prime },Z_{m^{S}}^{S}\right)\sum_{l=1}^{L}{\left(x_{\ell^{\prime }}^{l}-{\hat{x}}_{\ell^{\prime }}^{l}\left(Z_{m^{S}}^{S}\right)\right)}^{2}dx_{\ell^{\prime }}dz_{1:m^{S}}^{S} \end{array}$$

where the last line is obtained according to the marginal density $q_{n}\left(x,Z_{m^{S}}^{S}\right)$ of (25) and the definition of 2-norm, *L* is the dimension of x, $\int_{X_{n-1}}\cdot d\left(x_{1:n}-x_{\ell^{\prime }}\right)$ denotes the residual integral of $\int_{X_{n}}\cdot dx_{1:n}$ after the term $\int_{X_{1}}\cdot dx_{\ell^{\prime }}$ is separately excluded from $\int_{X_{n}}\cdot dx_{1:n}$.

Since **A2** has shown that the estimator ${\hat{x}}_{\ell^{\prime }}\left(Z_{m^{S}}^{S}\right)$ is unbiased, the information inequality of (7) can be applied to (A20),

(A21) $$\int_{{\mathbb{Z}}_{\hat{n},m^{S}}^{S}}\int_{X_{1}}q_{n}\left(x,\ell^{\prime },Z_{m^{S}}^{S}\right){\left(x_{\ell^{\prime }}^{l}-{\hat{x}}_{\ell^{\prime }}^{l}\left(Z_{m^{S}}^{S}\right)\right)}^{2}dx_{\ell^{\prime }}dz_{1:m^{S}}^{S}\begin{array}{cc} \geq {\left[J_{\hat{n},n,m^{S}}^{-1}(\ell^{\prime })\right]}^{l,l} & l=1,\ldots ,L \end{array}\;$$

where the FIM $J_{\hat{n},n,m^{S}}(\ell^{\prime })$ is shown in (42). (A21) holds with equality if and only if $q_{n}\left(x,\ell^{\prime },Z_{m^{S}}^{S}\right)$ satisfies the distribution of exponential family.

Substituting (A21) into (A20) and then (A19), we get

(A22) $$\begin{array}{ll} \sigma_{S}^{2} & \geq \sum_{m^{S}=0}^{\infty }\sum_{n=0}^{\infty }\sum_{\hat{n}=0,n+\hat{n}>0}^{\infty }\sum_{k=0}^{\min(n,\hat{n})}\sum_{{\ell^{\prime }}_{1:k}\in L_{k}}\frac{\Omega_{n,m^{S}}\Psi_{\hat{n},n,m^{S}}}{m^{S}!k!\left(n-k\right)!}\cdot \frac{1}{n+\hat{n}-k}\left(\sum_{\ell^{\prime }\in \{{\ell^{\prime }}_{1:k}\}}\min\left(c^{2},\frac{1}{\Psi_{\hat{n},n,m^{S}}}\sum_{l=1}^{L}{\left[J_{\hat{n},n,m^{S}}^{-1}(\ell^{\prime })\right]}^{l,l}\right)+c^{2}\left(n+\hat{n}-2k\right)\right)\\ & =\sum_{m^{S}=0}^{\infty }\sum_{n=0}^{\infty }\sum_{\hat{n}=0,n+\hat{n}>0}^{\infty }\sum_{k=0}^{\min(n,\hat{n})}\sum_{\ell^{\prime }\in L_{1}}\frac{\Omega_{n,m^{S}}\Psi_{\hat{n},n,m^{S}}}{m^{S}!\left(n-k\right)!}\cdot \frac{1}{n+\hat{n}-k}\left(k\begin{array}{c} \cdot \end{array}\min\left(c^{2},\frac{1}{\Psi_{\hat{n},n,m^{S}}}\sum_{l=1}^{L}{\left[J_{\hat{n},n,m^{S}}^{-1}(\ell^{\prime })\right]}^{l,l}\right)+c^{2}\left(n+\hat{n}-2k\right)\right) \end{array}$$

where the second line is derived from the fact that the FIM $J_{\hat{n},n,m^{S}}(\ell^{\prime })$ is the same for all $\ell^{\prime }\in \left\{{\ell^{\prime }}_{1:k}\right\}$ given ${\ell^{\prime }}_{1:k}\in L_{k}$.

Finally, (40) can be obtained by substituting the notation $\varepsilon_{k,\hat{n},n}$ defined in (41) into (A22). □.
